# Complete Proteome of a Quinolone-Resistant *Salmonella* Typhimurium Phage Type DT104B Clinical Strain

**DOI:** 10.3390/ijms150814191

**Published:** 2014-08-15

**Authors:** Susana Correia, Júlio D. Nunes-Miranda, Luís Pinto, Hugo M. Santos, María de Toro, Yolanda Sáenz, Carmen Torres, José Luis Capelo, Patrícia Poeta, Gilberto Igrejas

**Affiliations:** 1 Institute for Biotechnology and Bioengineering, Centre of Genomics and Biotechnology, University of Trás-os-Montes and Alto Douro, 5001-801 Vila Real, Portugal; E-Mails: scorreia@utad.pt (S.C.); j.dinis.miranda@utad.pt (J.D.N.-M.); lcpinto@utad.pt (L.P.); 2 Department of Genetics and Biotechnology, University of Trás-os-Montes and Alto Douro, 5001-801 Vila Real, Portugal; 3 Centre of Studies of Animal and Veterinary Sciences, University of Trás-os-Montes and Alto Douro, 5001-801 Vila Real, Portugal; E-Mail: ppoeta@utad.pt; 4 Veterinary Science Department, University of Trás-os-Montes and Alto Douro, 5001-801 Vila Real, Portugal; 5 BIOSCOPE group, REQUIMTE-CQFB, Chemistry Department, Faculty of Science and Technology, University NOVA of Lisbon, 2829-516 Monte de Caparica, Portugal; E-Mails: hms14862@fct.unl.pt (H.M.S.); jlcm@fct.unl.pt (J.L.C.); 6 Departamento de Biología Molecular (Universidad de Cantabria) and Instituto de Biomedicina y Biotecnología de Cantabria IBBTEC (UC-SODERCAN-CSIC), Santander 39011, Spain; E-Mail: detorom@unican.es; 7 Área de Microbiología Molecular, Centro de Investigación Biomédica de La Rioja, C/Piqueras 98, 26006 Logroño, La Rioja, Spain; E-Mails: ysaenz@riojasalud.es (Y.S.); carmen.torres@unirioja.es (C.T.); 8 Área de Bioquímica y Biología Molecular, Universidad de La Rioja, Av. Madre de Dios 51, 26006 Logroño, La Rioja, Spain

**Keywords:** *Salmonella enterica* serovar Typhimurium, DT104B, SL1344, proteome, aminoglycoside 6'-*N*-acetyltransferase type Ib-cr4

## Abstract

Salmonellosis is one of the most common and widely distributed foodborne diseases. The emergence of *Salmonella* strains that are resistant to a variety of antimicrobials is a serious global public health concern. *Salmonella enterica* serovar Typhimurium definitive phage type 104 (DT104) is one of these emerging epidemic multidrug resistant strains. Here we collate information from the diverse and comprehensive range of experiments on *Salmonella* proteomes that have been published. We then present a new study of the proteome of the quinolone-resistant Se20 strain (phage type DT104B), recovered after ciprofloxacin treatment and compared it to the proteome of reference strain SL1344. A total of 186 and 219 protein spots were recovered from Se20 and SL1344 protein extracts, respectively, after two-dimensional gel electrophoresis. The signatures of 94% of the protein spots were successfully identified through matrix-assisted laser desorption/ionization mass spectrometry (MALDI-TOF MS). Three antimicrobial resistance related proteins, whose genes were previously detected by polymerase chain reaction (PCR), were identified in the clinical strain. The presence of these proteins, dihydropteroate synthase type-2 (*sul*2 gene), aminoglycoside resistance protein A (*strA* gene) and aminoglycoside 6'-*N*-acetyltransferase type Ib-cr4 (*aac*(6')-*Ib-cr4* gene), was confirmed in the DT104B clinical strain. The *aac*(6')-*Ib-cr4* gene is responsible for plasmid-mediated aminoglycoside and quinolone resistance. This is a preliminary analysis of the proteome of these two *S.* Typhimurium strains and further work is being developed to better understand how antimicrobial resistance is developing in this pathogen.

## 1. Introduction

Non-typhoid *Salmonella* is a common and widely distributed cause of food poisoning [1]. Even though non-typhoid *Salmonella* frequently causes self-limited infections, some strains can also cause complicated invasive infections that require antimicrobial therapy [2]. The global burden of disease caused by *Salmonella* infections is substantial and the public health impact is aggravated by antimicrobial resistance, which leads to increased morbidity, mortality, and treatment costs [3]. Nowadays, *Salmonella* clinical isolates show high rates of resistance to traditional antimicrobials. Fluoroquinolones and expanded-spectrum cephalosporins have remained effective against non-typhoid *Salmonella* infection but resistance to these agents is also increasing [2]. Ciprofloxacin is an important last resort antimicrobial to treat complicated *Salmonella* infections because it can penetrate macrophages and eliminate multidrug-resistant strains [4]. Nevertheless, ciprofloxacin-resistant strains are becoming more common.

*Salmonella* is a prime model organism for infection biology research [5]. *S.* Typhimurium SL1344 is among the most extensively studied pathogenic strains and is frequently used as a reference organism to investigate *Salmonella* pathogenicity [6]. However, considering the high plasticity of bacterial genomes, the adequacy of laboratory-adapted reference strains for the study of “real-world” pathogenesis is being questioned [7]. As laboratory reference strains are repeatedly passaged *in vitro*, they can become significantly differentiated from clinical samples. Studies based on laboratory strains may therefore overlook important pathophysiological mechanisms that are only present in clinical strains [7].

*S*. Typhimurium phage type DT104 is an important multidrug-resistant clinical strain with an extensive host range that has been responsible for pandemic spread and many outbreaks over the last two decades [3,6]. Multiresistant DT104 strains were first isolated in the 1980s and commonly show resistance to ampicillin, chloramphenicol, streptomycin, sulfonamides and tetracycline (ACSSuT resistance type), with additional resistance to trimethoprim and ciprofloxacin [8]. Higher morbidity and mortality rates are likely to be associated with DT104 infections but it is not completely known why this particular strain has disseminated so successfully [6,8]. Recent studies have shown an emergence of hybrid virulence-resistance plasmids in *S.* Typhimurium DT104 that results from the integration of antimicrobial resistance genes into virulence plasmids involved in systemic infection [9]. These hybrid plasmids provide an adaptive advantage that enhances the epidemic potential of these strains.

Antimicrobial resistance and virulence are determinant in the clinical outcome of severe *Salmonella* infections, so it is important to understand how the associated genetic mechanisms are regulated [10]. Proteomics approaches can be used to investigate how genetic diversity can lead to the emergence of new resistance phenotypes and which protein interactions or post-translational modifications (PTM) are associated with antimicrobial resistance [11]. Genome mining in *Salmonella* showed that, due to its metabolic robustness, the number of potentially lethal targets for antimicrobial drug development is smaller than expected. Directly identifying bacterial proteins which prevent antibiotic resistance might expand the conventional armamentarium [12,13]. In the last decade, MS-based proteomics has been advancing rapidly, generating more information on functional and regulatory features. Proteomics results provide the most realistic depiction of infective processes because the methods detect the final products of gene biosynthetic pathways that truly define a biological phenotype [11,14].

Two dimensional gel electrophoresis (2-DE) is still one of the most powerful methods to study crude protein mixtures, as it is a selective, specific, reproducible, and reliable way to analyze several hundred proteins in a single experiment [15]. The analysis of bacterial proteomes can provide a global view of physiological adaptation, and 2-DE coupled with peptide mass fingerprinting (PMF) has been established as a standard tool to study diverse cellular functions and regulation [16]. For instance, total bacterial proteomes from different strains can be compared to identify proteins that correlate with different antimicrobial resistance profiles [17]. Table 1 sumarizes information from the many studies that have investigated *Salmonella* serotypes at the proteomic level.

In this work we investigated the complete proteomes of a clinical multidrug-resistant *S.* Typhimurium DT104B strain, designated as Se20 [18], and the reference *S.* Typhimurium SL1344 strain [19], in order to provide a snapshot of the major proteins involved in the basic cellular physiology of these strains, paying special attention to the expression of proteins related to antimicrobial resistance and virulence.

**Table 1:** List of *Salmonella* serotypes studied at the proteomic level with a short description of the main purpose and findings of each study.

Serotype	Strain	Main Purpose	Main Findings	Ref.
Typhimurium and Typhi	LT2 and Ty2	To perform a quantitative comparative proteomic analysis between *S.* Typhimurium and *S.* Typhi using SILAC coupled with LC-MS/MS.	Potential biomarker proteins with serovar-specific expression were identified. Flagella and chemotaxis genes were down-regulated in *S.* Typhi and proteins involved in metabolism and transport of carbohydrates and amino acids were differentially expressed.	[20]
Infantis	Soil isolate (from cattle manure)	To elucidate the global modulation of bacteria and plant protein expression after *Salmonella* internalization into lettuce.	Fifty proteins were differentially expressed between internalized and cutured *S.* Infantis. Internalized *S.* Infantis triggered the lettuce defense mechanisms. The bacteria might use ascorbate as a carbon source and require stress response proteins to cope with stresses incurred in plants.	[21]
Paratyphi A	YN07077, GZ9A0503, ZJ98053, ATCC 9150	To perform a 2-DE comparative proteomics analysis for 4 epidemic strains with different geospatial and temporal characteristics in order to obtain their core and pan proteomes.	The proteomes of the four strains were highly conserved. Few strain-specific proteins were found and non-core proteins were found in similar categories as core proteins. Significant fluctuations in the abundance of some core proteins suggest a variation in protein expression in the different strains even when cultured in the same conditions.	[22]
Typhimurium	ATCC 14028	To profile the intact proteome by single-dimension ultra-high-pressure liquid chromatography coupled with Velos-Orbitrap MS.	Identification of 563 proteins including 1665 proteoforms generated by PTMs. Report of a unique protein *S*-thiolation switch in response to infection-like conditions.	[23]
Typhimurium	ATCC 14028	To observe changes in protein abundance or location between phagosome-mimicking and standard laboratory conditions.	The protein subcellular localization of over 1000 proteins was catalogued. New insights into dynamic protein localization and potential moonlighting.	[24]
Typhimurium	ST23	To elucidate biocide tolerance mechanisms by comparing 2-D DIGE protein profiles of a triclosan sensitive strain and the isogenic tolerant mutant in the presence and absence of triclosan.	Triclosan exposure induced multiple changes in cellular metabolism, permeability, transport and also modifications involving mutations in the triclosan specific target FabI. Broader changes that may confer cross-resistance to antimicrobial agents were also observed.	[25]
Typhimurium	SL1344	To analyze differentially expressed proteins between a wild-type strain and an *opgGH* mutant to elucidate proteomic pleiotropy under low osmolarity.	The *opgGH* mutant had decreased protein amounts, consistent with the genotype and the expected phenotypes, and revealed pleiotropic proteome effects likely to enable survival under low-nutrient and low-osmotic growth conditions.	[26]
Typhimurium, Typhi and Choleraesuis	LT2, ATCC 33458 and SC-B67	To analyze the ability of pseudogenes to express normal protein sequences and to develop an experimental approach to detect recoding at the genomic scale using LC-MS/MS.	The majority of pseudogenes failed to express, validating the overall accuracy of *in silico* annotation. A few annotated pseudogenes translated normal peptides, suggesting that recoding may be common in bacterial species.	[27]
Gallinarum	9R and WT (287/91 and 06Q110)	To compare the proteome and transcriptome of wild-type and live vaccine strains of *S.* Gallinarum by 2-DE MALDI-TOF MS and microarray analysis.	One protein relevant to virulence absent from 9R. Analysis revealed 42 virulence genes down-regulated in the 9R transcriptome. The attenuation of 9R may be associated with a combination of impaired virulence factors so reversion to virulence is probably not caused by single mutation.	[28]
Enteritidis, Typhimurium and Gallinarum	Human and chicken isolates; 9R	To examine protein profile variability among *S.* Enteritidis, *S.* Typhimurium and *S.* Gallinarum by a comparative 2-DE MALDI-TOF MS proteomic analysis.	A high level of variation between serotypes was observed and several serotype-specific factors were detected. Proteins related to virulence, such as β-lactamase, RfbH protein, and shikimate kinase were identified.	[29]
Typhimurium	-	To characterize the proteome and ionome of wild type and znuA mutant strains under Zn starvation or Zn-replete conditions to gain further insight into Zn influx regulation.	Several differentially regulated proteins were predicted to be metal-binding proteins; their over-expression in the znuA mutant strain strictly depends on Zn starvation and correlates with differences found at the ionome level.	[30]
Typhimurium	VNP20009	To profile protein expression in the tumor-specific VNP strain under anaerobic and aerobic conditions, and to develop a hypoxia-inducible promoter system to confine expression of therapeutic genes within the tumor microenvironment.	The hypoxia-inducible *adhE* promoter was screened from the hypoxia-regulated endogenous proteins of *Salmonella* and proof-of-principle was provided that these promoter systems can be employed to target the hypoxic region of solid tumors and exert enhanced anticancer effects.	[31]
Typhimurium	ATCC 14028	To identify effector proteins secreted under SPI-2-inducing growth conditions using LC-MS/MS.	Eight novel effectors and ~80% of the previously reported ATCC14028 repertoire were identified including novel secreted effectors and new pathways for *Salmonella* virulence factors.	[32]
Typhimurium	SL1344	To identify post-transcriptional regulatory events by analyzing proteome changes after activation of the RcsCDB regulatory system.	Two new post-transcriptional regulatory processes were defined, inverse regulation by the *metE* and *pckA* genes and expression control of the small RNA FnrS by the RcsCDB system.	[33]
Typhimurium	MA6926	To survey the proteomic changes in response to low Mg^2+^ concentrations or CAMP in a SILAC-based quantitative proteomic approach.	CAMP activates a portion of the PhoP/PhoQ regulatory network. Low Mg^2+^ concentrations up-regulate nearly all known and some previously unknown members of this network, and also proteins regulated by IHF and RpoS.	[34]
Typhi	CT18	Characterization of anti-*S.* Typhi antibody responses in bacteremic Bangladeshi patients by immunoaffinity proteomics-based technology.	Identification of 57 proteins whose capture by affinity-purified antibody fractions from plasma of patients with *S.* Typhi bacteremia was significantly increased compared to the capture by the column without antibody.	[35]
Typhimurium	ATCC 14028	Proteome profiling of wild-type and mutant strains with ProteinChip arrays coupled to SELDI-TOF.	Revelation of differential regulation of the σ-dependent yciGFE(katN) locus by YncC and H-NS in *Salmonella* and *Escherichia coli* K-12.	[36]
Enteritidis	chicken isolate (LK5)	Global 2-DE MALDI-TOF MS protein analysis of *S.* Enteritidis adapted or unadapted to propionate.	The stress-related proteins Dps and CpxR5 were up-regulated in propionate-adapted cultures and play an important role in propionate-induced acid resistance.	[37]
Enteritidis	clinical isolate (SE2472)	To develop a stable isotope labeling procedure coupled with MS analysis to carry out quantitative proteomic analysis of *S.* Enteritidis upon exposure to hydrogen peroxide.	Identification of 76 proteins with H_2_O_2_ modulated expression. SPI-1 effector SipC was overexpressed and was found to be highly expressed in the spleen at late stage of *in vivo* infection, suggesting a role of SipC in supporting survival and replication under oxidative stress and during systemic infection *in vivo*.	[38]
Typhimurium and Enteritidis	wild boar and wild rabbit isolates	To determine and compare the proteomes of *S.* Typhimurium and *S.* Enteritidis recovered from faecal samples from wild boars and rabbits.	Different serotypes had different SDS-PAGE profiles. Proteins related to antibiotic resistance, pathogenesis and virulence were identified in both strains.	[39]
Typhimurium	LT2 (ATCC 700720)	To elucidate the expression of OMPs of *S.* Typhimurium using a LPI™ Flow-Cell lipid-based protein immobilization technique.	The LPI™ technique provided wide coverage with 54 OMPs identified, enabling the incorporation of a multi-step protease workflow that allows the identification of more membrane proteins with higher confidence.	[40]
Thompson	MCV1	To study the proteome changes of *S.* Thompson during stress adaptation to sublethal concentrations of thymol with 2-DE MALDI-TOF MS.	Several proteins from different functional classes were significantly up- or down-regulated showing that thymol plays a role in altering very different metabolic pathways.	[41]
Typhi and Typhimurium	Ty2, CT18, Ty800 and LT2	Comparative proteomic analysis to study PhoP/Q-dependent protein expression differences between *S.* Typhi and *S.* Typhimurium.	Identification of 53 PhoP-regulated proteins in LT2 and 56 in *S.* Typhi, including 3 *S.* Typhi-unique proteins (CdtB, HlyE and STY1499). First protein expression profile of the live attenuated bacterial vaccine studied in humans Ty800.	[42]
Typhimurium	clinical isolate and NCTC 74	To characterize proteins that are differentially expressed in the presence or absence of oxygen to reveal proteins that may allow the species to adapt and initiate infection in anaerobic conditions.	A drastic transformation in expression was observed with the shift to anaerobiosis. The responses of different isolates were not uniform and the high degree of change showed the potential limitation of using laboratory-grown strains to search for vaccine targets.	[43]
Typhimurium	DT104 (ATCC 700408)	To determine if protein profiling by GC-MS analysis of fatty acids with PCA and 2-DE can be used for rapid assessment and interpretation of the impact of SC-CO_2_ treatment.	SC-CO_2_ caused significant alterations in the fatty acid and protein profiles with 11 spots becoming more than 50% less intense. The low levels of the latter proteins may have negatively affected the survival of microbial cells.	[44]
Gallinarum and Enteritidis	JOL394	To discover host specificity and/or pathogenicity proteins among different host-adapted serovars by 2-DE MALDI-TOF MS/QRT-PCR analysis of serovar Gallinarum in comparison with Enteritidis.	In *S.* Gallinarum 22 proteins were over-expressed comparing to *S.* Enteritidis. Proteins were identified that are related to virulence or have unknown functions that may be important in the host adaptation and/or pathogenicity of *S.* Gallinarum.	[45]
Typhimurium	ATCC 14028	To investigate the macrophage response to infection by infecting RAW 264.7 macrophages and analyzing time course responses at the global proteomic level.	Identification of 1006 macrophage and 115 *Salmonella* proteins with high confidence. Most of the *Salmonella* proteins were observed in the late stage of infection, which is consistent with the fact that the bacterial cells proliferate inside RAW 264.7 macrophages.	[46]
Typhimurium	ATCC 14028	To determine the impact of a low Mg^2+^/pH defined growth medium (MgM) on the proteome of *S.* Typhimurium by a comparative LC-MS/MS approach.	MgM shock-induced proteins usually induced by low O_2_. MgM dilution induced the T3SS proteins SsaQ and SseE and also the biotin biosynthesis proteins BioB and BioD that also increased after infection of RAW 264.7 macrophages.	[47]
Typhimurium	SL1344	To investigate the role of AI-2/LuxS by a comparative 2D-DIGE analysis of wild type and a luxS mutant strain.	A few proteins were differentially expressed but further analysis of the LuxS protein revealed a PTM and a potential translocation across the cytoplasmic membrane.	[48]
Typhimurium	SL1344	To investigate the combined effect of low oxygen tension and high osmolarity on the proteome of *S.* Typhimurium compared to standard laboratory conditions by 2-D DIGE.	Under *in vivo*-like conditions anaerobic fumarate respiration and the utilization of 1,2-propanediol are up-regulated and an arginine deiminase pathway is expressed for l-arginine catabolism. Proteins involved in quorum sensing and virulence are also differentially expressed.	[49]
Typhimurium	SL1344	To determine and compare the proteomes of three triclosan resistant mutants to identify proteins involved in triclosan resistance.	Proteins involved in pyruvate or fatty acid production were differentially expressed in all mutants. Triclosan resistance is multifactorial and several resistance mechanisms act in synergy to achieve high-level resistance.	[50]
Typhimurium	ATCC 13311	Characterization of the OMP-immunoreactive fractions in *Salmonella* induced reactive arthritis by SDS-PAGE and MALDI-TOF MS.	Identification of 10 low molecular weight OMPs which are T-cell immunoreactive in patients with *Salmonella* induced reactive arthritis/undifferentiated spondyloarthropathy.	[51]
Typhimurium	01-45, R200 and 6B7	To compare OMP profiles between a *yjeH* mutant with reduced resistance to ceftriaxone and the resistant parental strain, by 2-DE MALDI-TOF MS/MS.	*yjeH* gene inactivation resulted in a 4-fold reduction in ceftriaxone resistance and in an underexpression of STM1530, STM3031, MopA, and NuoB, but overexpression of OmpD. Expression of the *S.* Typhimurium *yjeH* gene also confers ceftriaxone resistance in *E. coli*.	[52]
Typhimurium	CS022	To compare a proteome defined by shotgun proteomics directly on an LTQ-FT and by proteome pre-fractionation on an LCQ-DUO.	Shotgun proteomic analyses on the LCQ-DUO adequately characterized a PhoP constitutive strain if proteome pre-fractionation steps and gas-phase fractionation were included.	[53]
Typhimurium	STM14028	To identify key proteins linked to macrophage colonization by LC-MS analysis of protein abundance in *Salmonella* cells isolated from RAW264.7 macrophages, with or without functional Nramp1, at various time points of infection.	After infection 39 proteins were strongly induced, 6 of which are modulated by Nramp1, including STM3117. Deletion of the STM3117 gene caused a dramatic reduction in the ability to colonize macrophages, demonstrating that STM3117 is an important virulence factor that promotes replication inside macrophages.	[54]
Typhimurium	SL1344	To investigate the physiological response of *S.* Typhimurium to fluoroquinolone antibiotics by 2-DE and 2D-LC-MS.	Several proteins were over or underexpressed. An increase in AcrAB/TolC was associated with resistance while F1F0-ATP synthase and Imp increased in response to fluoroquinolones.	[55]
Typhimurium	ATCC 14028 and LT2	To analyze the *S.* Typhimurium proteome under laboratory and infection-like conditions through a LC-MS-based “bottom-up” proteomic approach.	A comprehensive view of protein abundances as they vary with respect to time, environment, and genotype. Results support earlier observations that *pdu* gene expression contributes to *S.* Typhimurium pathogenesis.	[56]
Typhimurium and Pullorum	NCTC 74, 4 clinical isolates, A01, C01; NCTC 10704, B52	To compare the expression patterns of host restricted *S.* Pullorum and host generalist *S.* Typhimurium isolates with a combined 2-DE LC-MS/MS proteomic approach.	Isolates varied greatly and, in some cases, more between the same serotype than between different serotypes. New serotype-specific proteins were identified, including intermediates in sulphate utilization and cysteine synthesis.	[57]
Typhi	clinical isolate (5866)	Analysis of the pleiotropic effects of a deficiency in the periplasmic disulfide-bond oxidoreductase DsbA using 2-DE MALDI-TOF MS.	In total, 25 spots were exclusive to the wild-type strain, 10 to the *dsbA*-null mutant, and 21 were common to both. DsbA, glucose-1-phosphatase, flagellin and the AI-2 autoinducer-producing LuxS were absent in the *dsbA*-null mutant.	[58]
Typhimurium	SL1344	Proteome characterization by 2D-HPLC MS to provide a platform for subsequent proteomic studies of low level multiple antibiotic resistance.	A total of 34 OMPs were detected and 20 proteins previously associated with the *mar* locus in *E. coli* were also identified including the key MAR effectors AcrA, TolC and OmpF.	[59]
Typhimurium	UK1 (WT) and RJ1827	To compare changes in gene expression caused by *fis* mutation through a 2-DE MS proteomic approach in order to elucidate the role of Fis in *Salmonella* virulence.	Identification of 11 proteins upregulated and 7 downregulated by Fis, involved in translation, sugar metabolism, flagellar synthesis, and virulence. Changes in SPI expression suggest that gene regulation in SPI-2 and in SPI-1 is affected by Fis.	[60]
Typhimurium	ATCC 14028	To identify low-level expressed proteins by expressing several SPI2 T3SS putative proteins as recombinant products followed by by 2-DE MALDI-MS detection.	Recombinant expression is a complementary tool to analyze low abundant or membrane proteins. Pre-fractionation and pulse labeling allowed the identification of growth phase regulated SPI2 proteins that might not be detected otherwise.	[61]
Typhimurium	SL1344	To identify acid-regulated elements of the flagellar system and to study how they are regulated by low pH.	Flagella-mediated cell motility is co-regulated by low pH via the PhoPQ signal transduction system.	[62]
Typhimurium	SL1344	To test the feasibility of proteome determination by identifying 53 randomly sequenced cell envelope proteins by *N*-terminal sequencing of spots from 2D gels.	The existence of previously hypothetical proteins predicted from genomic sequencing projects was confirmed, and approximately 20% of the proteins had no matches in sequence databases.	[63]
Typhimurium	SL1344	To present a 2D reference map for proteins of the cell envelope fraction of *S.* Typhimurium SL1344.	In total 49 proteins were identified by microsequencing and assigned to a 2D reference map. Of these, 10 proteins seem to be new and others closely match putative proteins or proteins found in other bacteria but not previously reported in salmonellae.	[64]

2-D DIGE, two-dimensional difference gel electrophoresis; CAMP, cationic antimicrobial peptides; GC-MS, gas chromatography—mass spectrometry; LC-MS/MS, liquid chromatography coupled with tandem mass spectrometry; OMPs, outer membrane proteins; PCA, principal component analysis; QRT-PCR, quantitative real time—PCR; SC-CO_2_, supercritical carbon dioxide; SDS-PAGE, sodium dodecyl sulfate—olyacrylamide gel electrophoresis; SELDI-TOF, surface-enhanced laser desorption/ionization-time of flight; SILAC, stable isotope labeling by amino acids in cell culture; SPI, *Salmonella* pathogenicity island; T3SS, type three secretion system.

## 2. Results and Discussion

The proteomes of two *S.* Typhimurium strains, a multidrug-resistant phage type DT104B clinical strain (Se20) [18] and the phage type DT44 reference strain SL1344 [19,65], grown under standard culture conditions, were determined by 2-DE and MALDI-TOF MS identification.

**Figure 1:**
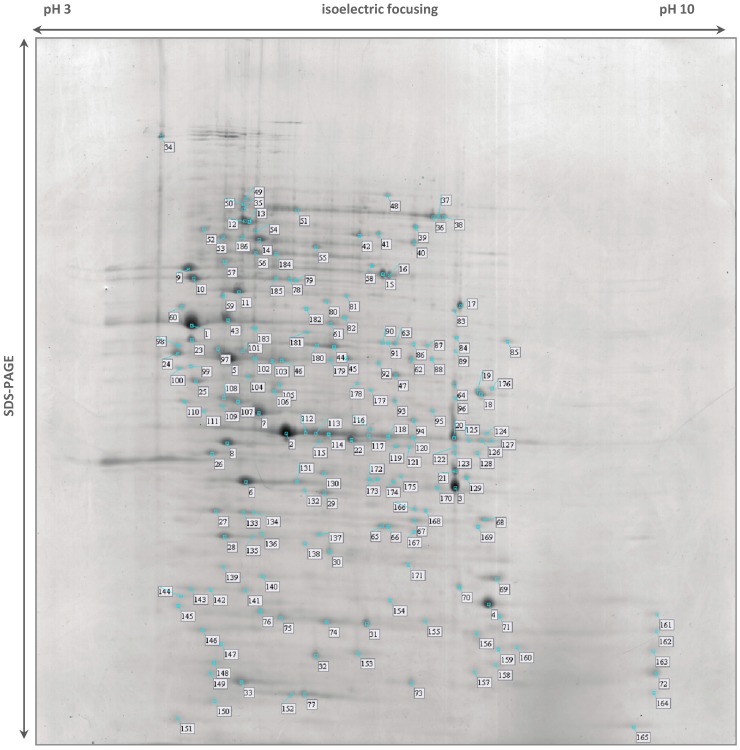
Stained 2-DE (two dimensional gel electrophoresis) gel of total proteins of *Salmonella* Typhimurium Se20 (phage type DT104B) using IPG (Immobiline™ pH Gradient) strips pH 3–10 NL (non-linear) for the first dimension. Numbered spots were excised for analysis by in-gel digestion and MALDI-TOF MS (matrix-assisted laser desorption/ionization mass spectrometry) identification, described in Table S1.

**Figure 2:**
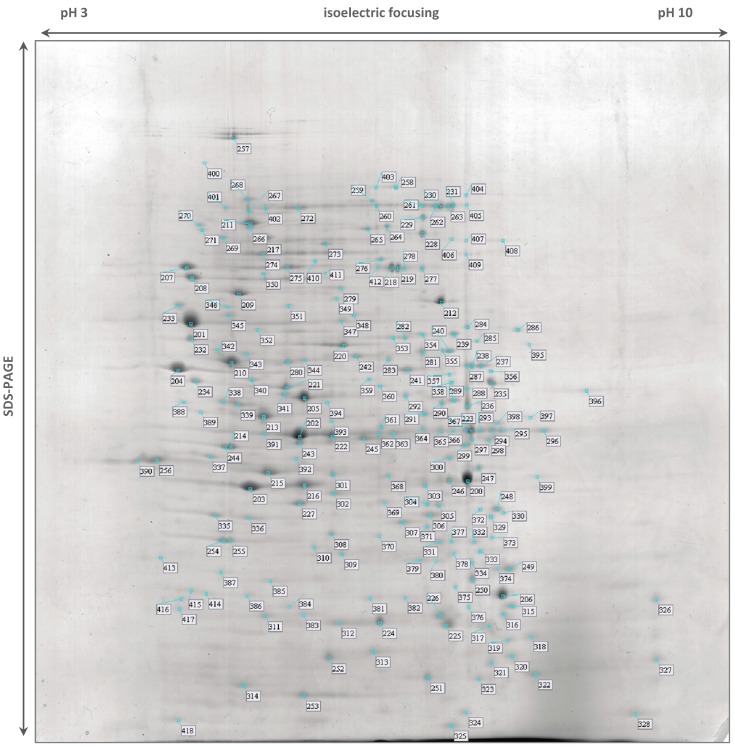
Stained 2-DE gel of total proteins of *Salmonella* Typhimurium SL1344 using IPG strips pH 3–10 NL for the first dimension. Numbered spots were excised for analysis by in-gel digestion and MALDI-TOF MS identification, described in Table S2.

**Figure 3:**
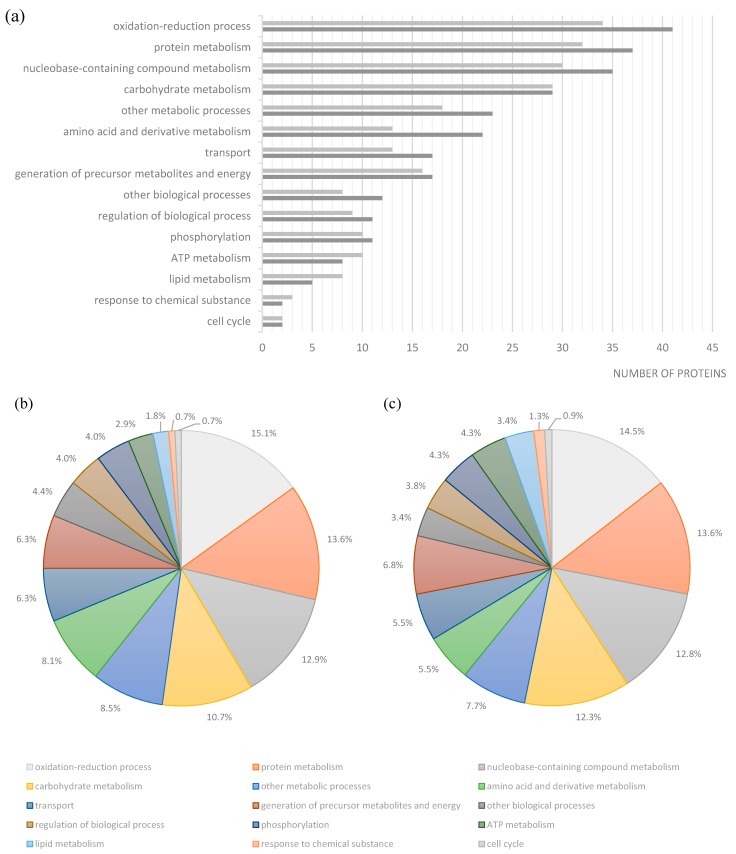
Functional classification of proteins identified in the Se20 and SL1344 strains based on Gene Ontology. (**a**) Number of proteins in each category for Se20 (light gray) and SL1344 (dark gray); Relative percentages of protein functions in (**b**) Se20 and (**c**) SL1344. As this classification reflects the fact that single proteins can be involved in more than one process, the sum of proteins in all categories is higher than the total number of unique proteins identified.

The *S.* Typhimurium DT104B clinical strain analyzed in this study was recovered from an elderly patient hospitalized with acute gastroenteritis and treated with ciprofloxacin. *In vivo* selection of quinolone and aminoglycoside resistance was observed post-treatment [18]. This strain was resistant to nalidixic acid, to all of the fluoroquinolones tested (ciprofloxacin, levofloxacin, ofloxacin and norfloxacin) and to the aminoglycosides amikacin, tobramycin, kanamycin and streptomycin. Strain Se20 was also resistant to tetracycline, trimethoprim/sulfamethoxazole, sulfonamides, trimethoprim and fusidic acid [18]. This strain harbored the antimicrobial resistance genes *tet*(A), *str*A, *str*B and *sul*2 and the plasmid-mediated quinolone resistance genes *qnr*S1 and *aac*(6')-*Ib-cr4*. The S83Y substitution in GyrA, which confers quinolone resistance, was also detected [18,66]. The virulent *S.* Typhimurium SL1344 reference strain was originally isolated from a calf with salmonellosis and is resistant to streptomycin and sulfonamide antimicrobials [19,67,68]. In this work, we recovered 186 protein spots from the 2-DE gel of strain Se20 (Figure 1) and 219 spots from strain SL1344 (Figure 2). After MALDI-TOF MS analysis, a total of 178 (96%) proteins representing 143 unique gene products were identified in strain Se20 (Table S1) and 202 (92%) proteins representing 166 unique gene products were identified in strain SL1344 (Table S2). The Gene Ontology (GO) annotations database was used to search for the biological processes assigned to each protein. A clustering algorithm (simRel) relying on semantic similarity measures was used to reduce the redundancy of GO terms using the web server tool REViGO [69] and a broad overview of the gene product attributes was achieved by using a GO slim based on the generic GO slim developed by the GO consortium. Approximately 50% of the proteins identified in both strains were related to oxidation-reduction processes, protein metabolism (chemical reactions and pathways involving a specific protein, including protein modifications), nucleobase-containing compound metabolism (processes involving nucleobases, nucleosides, nucleotides and nucleic acids) and carbohydrate metabolism (Figure 3). Table 2 indicates some relevant proteins that were exclusively identified in each of the studied strains.

**Table 2:** List of some relevant proteins exclusively identified either in *Salmonella* Typhimurium strain Se20 or in *Salmonella* Typhimurium strain SL1344.

Strain	Spots	Protein	Gene	Biological Process
Se20	24/98	Flagellin	fljB	ciliary or bacterial-type flagellar motility
	75	aminoglycoside 6'-*N*-acetyltransferase type Ib-cr, AAC(6')-Ib-cr4	aac(6')-Ib-cr4	metabolic process
	99	ethanolamine ammonia-lyase heavy subunit	eutB	cellular amino acid metabolic process
	106	ATP-dependent protease	hslU	ATP catabolic process, proteolysis, response to stress, protein unfolding
	134	universal stress protein E	uspE	response to stress
	142/143	aminoglycoside resistance protein A	strA	response to antibiotic
	148	Chain E, Alkyl Hydroperoxide Reductase C (Substrate-Ready Conformation)	ahpC	response to oxidative stress, oxidation-reduction process
	182	5'-nucleotidase	ushA	dephosphorylation, nucleotide catabolic process
SL1344	205/341	arginine deiminase	arcA	protein citrullination
	215	ornithine carbamoyltransferase	arcB	ornithine metabolic process
	225	fumarate reductase iron-sulfur subunit	frdB	tricarboxylic acid cycle
	227	carbamate kinase	arcC	arginine metabolic process
	237/238/287	glycerol-3-phosphate dehydrogenase	glpD	glycerol-3-phosphate metabolic process
	240	inosine 5'-monophosphate dehydrogenase	guaB	purine nucleotide biosynthetic process
	259	NADH dehydrogenase subunit G	nuoG	ATP synthesis coupled electron transport
	296	molecular chaperone DnaJ	dnaJ	response to stress
	332	Hydrogenase	-	-
	344	Phosphoglucomutase	pgm	carbohydrate metabolic process
	346	oligopeptidase A	prlC	proteolysis
	378	exonuclease III	xth	DNA catabolic process, exonucleolytic
	396	serine endoprotease	htrA	proteolysis
	406	cell invasion protein SipA	sipA	pathogenesis

When comparing the proteins identified in each of the analysed strains, it is important to refer the exclusive presence of the aminoglycoside 6'-*N*-acetyltransferase type Ib-cr4 (AAC(6')-Ib-cr4) protein (spot 75) and the aminoglycoside resistance protein A (spots 142 and 143) in the Se20 clinical strain (Figure 1, Table S1). These two proteins reflect the antimicrobial resistance phenotype observed in Se20 for the aminoglycosides amikacin, tobramycin and kanamycin and for the fluoroquinolones ciprofloxacin, levofloxacin, ofloxacin and norfloxacin. The AAC(6')-Ib-cr4 protein is encoded by the *aac*(6')-*Ib-cr4* gene, which was previously detected in this strain on plasmid pMdT1 [18,66]. The 225 amino acid protein here detected was predicted by ORF (open reading frame) Finder analysis to have a longer *N*-terminal length when comparing to other previously described functional aac(6')-Ib variants [66]. The position of spot 75 matches the theoretical molecular weight (MW) of 25031 Da and isoelectric point (pI) value of 5.2 estimated for AAC(6')-Ib-cr4. The AAC(6')-Ib-cr protein is a variant of the widespread aminoglycoside acetyltransferase AAC(6')-Ib that is usually responsible for resistance to amikacin, kanamycin and tobramycin. The AAC(6')-Ib-cr variant also acetylates ciprofloxacin and norfloxacin, but less efficiently than aminoglycoside substrates [70]. Acetylation occurs at the amino nitrogen on the piperazinyl substituent, so only fluoroquinolones with an unsubstituted piperazinyl group are substrates of AAC(6')-Ib-cr. Even though the presence of the *aac*(6')-*Ib-cr* gene confers only low-level resistance to substrate fluoroquinolones, it may facilitate the survival of target-site mutants with a 10-fold increase in their mutant prevention concentration [71].

The aminoglycoside resistance protein A, coded by the previously detected *str*A gene, is an aminoglycoside 3'-phosphotransferase that catalyzes the transfer of the gamma-phosphoryl group from ATP to aminoglycoside antimicrobials, inactivating them [72]. Theoretically this protein has a *M*_W_ of 30,474 Da and a pI value of 4.7. In the 2-DE gel, the two corresponding spots have a *M*_W_ similar to the theoretical value, however spot 142 is slightly more basic than spot 143 (Figure 1). Single proteins separated by 2-DE frequently exhibit multiple spots in the first dimension. These so-called “charge trains” can be caused by isoform differences and post-translational modifications (PTMs). Some PTMs, such as phosphorylation, deamidation, desulfuration or acylation, can lead to electrical charge heterogeneity with minor modifications in molecular weight. Cysteine oxidation has also been reported to be responsible for pI basic shifts [15]. Nontheless, “charge trains” can also be considered artifacts due to the sample treatment and analytical procedures employed, such as carbamylation in the presence of urea or acrylamide adduct formation during electrophoresis [73].

The two strains analysed in this study present phenotypic resistance to sulphonamides. The target of sulfonamide antimicrobials and the basis for their selective effect on bacteria is dihydropteroate synthase (DHPS) in the folic acid pathway [74]. DHPS is a functional homodimer that, in prokaryotes, catalyzes the condensation of *p*-aminobenzoic acid (PABA) in the *de novo* biosynthesis of folate, an essential cofactor in protein and nucleic acid biosynthesis [72]. Higher eukaryotes are able to utilize dietary folate, so they do not have DHPS enzymes. Sulfonamides act either by competitively inhibiting DHPS by structural similarity with the PABA substrate or by functioning as alternative substrates for DHPS, forming pterin adducts that are unable to participate in folate biosynthesis [74]. DHPS was identified in both Se20 (spot 154, Figure 1, Table S1) and SL1344 (spot 382, Figure 2, Table S2) strains. In enteric Gram-negative bacteria, sulfonamide clinical resistance is plasmid-mediated by genes such as *sul*1 and *sul*2, which encode alternative drug-resistance variants of DHPS that show high insensitivity to sulfonamide drugs but bind normally to the PABA substrate [74]. The DHPS identified in this study (AC: S5HED7) is plasmid-encoded and shows a 100% sequence identity with the *S.* Typhimurium SL1344 DHPS type-2 (AC: H8WV44), which is present on the pRSF1010^SL1344^ plasmid. The *sul*2 gene, which encodes the type-2 DHPS, was previously reported in the Se20 strain [18] and also in the SL1344 strain [75].

Several other proteins related to antimicrobial resistance or virulence were identified. The outer membrane protein (OmpA) is one of the main surface proteins in Enterobacteriaceae species and has essential roles in the maintenance of structural cell integrity, transmembrane ion transport, mammalian cell invasion, bacteriophage binding, and conjugation [76]. OmpA was detected in two different gel locations in both strains. The more abundant spots, 6 for Se20 (Figure 1) and 203 for SL1344 (Figure 2), were found where expected for proteins with theoretical *M*_W_ of 37,606 Da and pI of 5.5. The less abundant spots, 141 for Se20 (Figure 1) and 386 for SL1344 (Figure 2), had the same pI but a lower *M*_W_ of approximately 30 kDa. However, these results are not unexpected as OmpA is known to run anomalously in SDS-PAGE [76]. β-barrel structures of bacterial outer membrane proteins are usually very stable and survive the SDS denaturing treatment at room temperature. As a result, native and denatured forms of many OMPs migrate at two different apparent molecular weights in SDS-PAGE. The OmpA protein was previously reported to migrate at 30 kDa in its native compacted form [77].

The porin outer membrane protein C (OmpC) was also identified. Antimicrobials such as ciprofloxacin, norfloxacin, cefepime and ceftriaxone strongly interact with OmpC, and so their translocation through this channel is facilitated [78]. The ion channel protein Tsx, which is also likely to play a role in antimicrobial resistance [59], was also identified. TolB, a periplasmic protein associated with the outer-membrane protein Pal, was detected. TolB belongs to the Tol-Pal system that is well conserved among Gram-negative bacteria and plays several roles, including lipopolysaccharide *O*-antigen surface expression, outer membrane stability, uptake of filamentous phage DNA, resistance to detergents and virulence [79].

The majority of the proteins identified in this study are involved in oxidation-reduction processes (Figure 3). One of the proteins identified in this class was the alkyl hydroperoxide reductase subunit C (AhpC), also named alkyl hydroperoxide reductase protein C22 (spot 148). In bacteria, this enzyme is responsible for hydrogen peroxide removal, a response to oxidative stress. The peroxide-reducing activities of AhpC help to protect pathogenic bacteria from the host immune response [80]; therefore the identification of this protein in the Se20 strain is in accordance with its host-adapted phenotype. The AhpC protein has recently been considered as a possible target for the development of new antimicrobial agents [80]. Other stress response proteins were also identified, namely the heat shock chaperone proteins DnaK, DnaJ, HtpG, HslU, HtrA, GroL, the protein disaggregation chaperone and the universal stress protein E (UspE). Another heat shock protein identified was the Lon protease (HAMAP-Rule MF_01973), which is required for cellular homeostasis and for survival from DNA damage and developmental changes induced by stress.

Bactericidal antimicrobials can induce cell death by stimulating the production of reactive oxygen species, principally O_2_^−^, which induces oxidative damage [81]. Superoxide dismutases are responsible for the destruction of these superoxide anion radicals. In addition to their detoxifying function, bacterial superoxide dismutases have also been shown to be important virulence factors [82]. In *S.* Typhimurium, SodA and SodB are cytoplasmic superoxide dismutases that require manganese and iron respectively as cofactors [83]. Some studies show that the expression of superoxide dismutase enzymes increases in response to antimicrobial stress [81]. Here, both cytoplasmic superoxide dismutases, SodA (AC: P43019) and SodB (AC: P0A2F5), were identified.

Another important virulence factor that shows significant expression in the Se20 clinical strain is flagellin, identified in spots 24 and 98. A tight regulation of flagella expression is essential for *Salmonella* when interacting with the host. Flagella-mediated virulence can be activated in the early stage of infection to increase invasiveness and can after be deactivated in order to minimize flagellin recognition by the host innate immune system and therefore prevent flagella-associated vulnerabilities. More than a motility associated virulence factor, flagella have a role in biofilm formation, are essential for *in vivo* multiplication, confer an advantage in the early stage of infection allowing rapid invasion of host cells, and also activate the host immune system while inactivating epithelial cell apoptosis [84]. Individual *Salmonella* serotypes usually alternate between the production of two antigenic forms of flagella, phase I and phase II, each specified by separate structural genes, *fliC* and *fljB*. Our results show that although the phase II flagellin seems to be higly expressed in the Se20 clinical strain and absent from SL1344, the phase I flagellin middle domain variant C12 was identified in both strains (spot 100 and spot 204), and shows a considerable higer expression in the SL1344 reference strain.

An additional protein identified in the clinical strain that may contribute to the pathogenic phenotype of DT104 is the ethanolamine ammonia-lyase (spot 99). Ethanolamine can be readily derived from cell membranes and therefore is available in the large intestine due to enterocyte turnover. Some bacteria, including *Salmonella*, are able to use ethanolamine as a source of carbon and/or nitrogen in a process that involves the conversion of ethanolamine into acetaldehyde and ammonia by an ethanolamine ammonia lyase [85]. Evidence was provided that in the inflamed intestine, *S*. Typhimurium has a growth advantage due to its ability to respire ethanolamine that is not utilizable by competing bacteria, showing a direct link between ethanolamine utilization and bacterial pathogenesis [86].

Further, concerning the Se20 clinical strain, the 5'-nucleotidase UDP (uridine diphosphate)-sugar hydrolase (UshA), was identified in spot 182, and appears to be absent in the reference strain. In *Escherichia coli*, UshA has an important function in nucleotide salvage. However, UshA can also function as a phosphate starvation-induced 5'-nucleotidase, being required for growth when nucleotides are provided as the only source of phosphate [87]. This condition is likely to be significant for bacterial growth in the wild [87], which may play a role in the worldwide dissemination on this strain.

The majority of proteins identified exclusively in SL1344 also reflect the virulence characteristics of this strain. The proteins arginine deiminase (ADI), ornithine carbamoyltransferase and carbamate kinase, constitute the ADI system that, besides its metabolic functions, has also been associated with virulence in some pathogens. These three proteins were identified in the high intensity spots 205, 215 and 227, respectively. It was previously established that the ADI pathway contributes to *Salmonella* pathogenesis and that arginine deiminase activity has an active role in the successful infection of mammalian hosts by *S.* Typhimurium [88].

Another high intensity spot was identified as the fumarate reductase iron-sulfur subunit (spot 225). A recent study provided evidence that fumarate reductase is associated with the bacterial flagellar switch complex, which determines the direction of flagellar rotation and is essential for chemotaxis. Fumarate influences the interaction of fumarate reductase with the FliG switch thus affecting flagellar assembly and rotation [89].

The study of specific proteins participating in *de novo* purine synthesis have shown that the absence of key enzymes in the pathway, namely the inosine 5'-monophosphate dehydrogenase GuaB, can severely attenuate growth rates and directly affect virulence in *S.* Typhimurium [90]. GuaB was only identified in spot 240 of SL1344.

Exonuclease III (spot 378), is an intermediate in the second step of the base excision repair (BER) system of oxidatively damaged DNA. *S.* Typhimurium suffers an oxidative DNA damage within macrophages that is repaired by the BER system. Hence, a functional BER system is required for *Salmonella* intramacrophage survival and contributes to systemic *Salmonella* infection [91].

The NADH dehydrogenase I coded by the *nuoG* gene (spot 259) is induced under microaerophilic and stationary-phase growth conditions. Mutations in *nuo* genes affect several mechanisms of microbial physiology and biochemistry which have direct consequences in *Salmonella* virulence [92].

Hydrogenase, identified in spot 332, has also been described to be essential to virulence in *S.* Typhimurium. The usage of respiratory hydrogen as a critical growth substrate for energy production allows colonization of the animal host and subsequent virulence during infection. Therefore, hydrogenases can represent potential therapeutic targets to combat *Salmonella* infections [93].

The phosphoglucomutase enzyme (spot 344) is important in the virulence of numerous pathogens and was recently reported to be required by *S.* Typhimurium for *O*-antigen production, resistance to antimicrobial peptides and *in vivo* fitness [94]. Oligopeptidase A, which is involved in degradation of signal peptides after they are released from precursor forms of secreted proteins, is also a virulence factor and heat shock protein that was identified only in the reference strain (spot 346) [95].

The effector SipA protein identified in SL1344 spot 406 is secreted by the centisome 63 type III secretion system encoded by *Salmonella* pathogenicity island 1 and is known to be a key factor in the invasion of epithelial cells by *S.* Typhimurium [96].

Finally, the glycerol-3-phosphate dehydrogenase GlpD was identified in spots 237, 238 and 287 of the SL1344 strain. In a recent study in *E. coli*, GlpD overexpression resulted in high persisters, *i.e.*, in a bacterial subpopulation capable of surviving antimicrobial exposure or other lethal treatments [97].

Effective therapies to treat resistant bacteria are urgently needed. We must understand the mechanisms underlying antimicrobial drug resistance in more detail, as no single bacterial strain can truly represent its species [7]. In this proteomic analysis we provide a physiological map and an overview of global protein expression of *Salmonella* Typhimurium Se20 (phage type DT104B) and SL1344 strains under normal growth conditions [14].

## 3. Experimental Section

### 3.1. Bacterial Strains and Growth Conditions

Two strains of *S.* Typhimurium, Se20 [18] and SL1344 [19], were included in this study. Se20 (phage type DT104B) is a previously characterized strain that was recovered from a faecal sample of an elderly patient who was admitted to a Spanish hospital with acute gastroenteritis. The patient was treated for 7 days with ciprofloxacin, and *in vivo* selection of quinolone resistance was observed post-treatment [18]. Frozen cell stocks of *S.* Typhimurium Se20 and SL1344 were streaked onto LB (Luria-Bertani) agar (Miller, Scharlau Chemie, S*.*A. Barcelona, Spain) plates and grown overnight at 37 °C. Pre-cultures were prepared by inoculation of 10 mL of LB broth (Miller, Scharlau Chemie, S.A.) with single colonies of each strain with further overnight incubation at 37 °C. Pre-cultures were diluted to an optical density at 600 nm (OD_600_) of 0.02 in a final volume of 10 mL of LB broth, and incubated at 37 °C for 5 h.

### 3.2. Protein Extraction

Cultures were harvested in the late exponential phase (OD_600_ of 0.5) by centrifugation at 10,000× *g* for 3 min at 4 °C and washed by centrifugation with 4 mL of phosphate-buffered saline (PBS). Bacterial cell pellets were suspended in 0.2 mL of solubilization buffer (10% (*w*/*v*) SDS and 12% (*w*/*v*) Tris) and lysed by sonication (4 × 10 s, 20 kHz, 100 W) at 4 °C. Cell debris were removed by centrifugation at 14,000× *g* for 30 min at 4 °C and proteins were further precipitated with cold trichloroacetic acid (TCA) at a final concentration of 20%. The proteins were recovered by centrifugation at 15,000× *g* for 25 min at 4 °C and washed twice by centrifugation in 0.3 mL of cold acetone for 10 min. Protein pellets were left to air-dry at room temperature. Proteins were extracted from three independent cultures andquantified by the Bradford method [98].

### 3.3. Two-Dimensional Gel Electrophoresis

Two-dimensional gel electrophoresis (2-DE) was performed according to the principles of O’Farrell [99] but with Immobiline™ pH Gradient (IPG) technology [100]. For isoelectric focusing, precast 13-cm IPG strips with a non-linear gradient from pH 3 to pH 10 (pH 3–10 NL, Amersham Biosciences, GE Healthcare, Uppsala, Sweden) were passively rehydrated overnight (16 h) at room temperature in a reswelling tray with 250 μL of rehydration buffer (8 M urea, 1% CHAPS (3-[(3-cholamidopropyl)-dimethylammonio]-propane-sulfonate), 0.4% DTT (dithiothreitol), 0.5% carrier ampholyte IPG buffer pH 3–10), covered with Dry-Strip Cover Fluid (Plus One, Amersham Biosciences, GE Healthcare). The protein samples (100 μg) were cup-loaded onto the rehydrated IPG strips [101] and focused at 500 V for 1 h, 1000 V for 8 h, 8000 V for 3 h and finally 8000 V incremented to 21,881 Vh in an Ettan™ IPGPhor II™ apparatus (Amersham Biosciences, GE Healthcare). Before the second dimension of electrophoresis, the focused IPG strips were equilibrated twice, each time for 15 min as follows. For the first equilibration, 1% DTT was added to equilibration stock buffer (6 M urea, 30% (*w*/*v*) glycerol, 2% (*w*/*v*) SDS in 0.05 M Tris–HCl buffer pH 8.8) and in the second equilibration, 4% iodoacetamide was added to equilibration stock buffer. Bromophenol blue was also added to both solutions. The equilibrated IPG strips were then gently rinsed with SDS electrophoresis buffer, blotted to remove excessive buffer, and then applied to 12.52% polyacrylamide gels in a Hoefer™ SE 600 Ruby^®^ (Amersham Biosciences, GE Healthcare) unit. The Laemmli SDS-PAGE technique was used with some modifications [102]. After the second dimension of separation, the 2-DE gels were fixed in a 40% methanol/10% acetic acid solution for 1 h with agitation, then stained overnight in Coomassie Brilliant Blue G-250 with agitation [103]. Gels were rinsed twice with 40% methanol for 45 min to remove excess staining and scanned on a flatbed scanner (Umax PowerLook 1100, Fremont, CA, USA). At least three 2-DE gels were run per protein sample. Images were analyzed using Image Master 5.0 software (Amersham Biosciences, GE Healthcare).

### 3.4. Tryptic Digestion of In-Gel Proteins

Coomassie Blue stained protein spots were manually excised and destained with 50% acetonitrile (ACN) in 25 mM ammonium bicarbonate. Gel pieces were dehydrated in neat ACN and dried under vacuum centrifugation. Enough trypsin solution (0.02 μg/μL), usually 15 μL, was added to cover each dried gel piece which was left on ice to rehydrate and to allow enzyme diffusion into the gel matrix. After 1 h, any solution not absorbed was removed and 15 μL of 12.5 mM ammonium bicarbonate was added, to immerse the gel piece. Proteins were digested overnight at 37 °C. The enzymatic reaction was stopped with 25 μL of 5% formic acid solution and the liquid mixture was collected. Finally, 25 μL of 50% (*v*/*v*) ACN/0.1% (*v*/*v*) TFA (trifluoroacetic acid) solution was added to the remaining gel pieces to increase the recovery of peptides. The extracted fractions were combined and dried in a Speed-Vac.

### 3.5. Peptide Mass Fingerprinting

Prior to protein digest analysis, each tryptic peptide mixture was ressuspended in 10 μL of 0.3% formic acid. Then, 1 μL of the resuspension was hand-spotted onto a MALDI target plate (384-spot ground steel plate), overlaid with 1 μL of α-cyano-4-hydroxycinnamic acid matrix solution (7 mg/mL in 0.1% (*v*/*v*) TFA/50% (*v*/*v*) ACN/8 mM ammonium phosphate) and dried under ambient conditions. All mass spectra were generated on a MALDI-TOF/TOF mass spectrometer Ultraflex (Bruker Daltonics, Bremen, Germany), operating in positive ion reflectron-mode. Spectra were acquired in the *m*/*z* range of 600–3500. A total of 500 spectra were acquired for each sample at a laser frequency of 50 Hz. External calibration was performed with the [M + H]^+^ monoisotopic peaks of bradykinin 1–7 (*m*/*z* 757.3992), angiotensin II (*m*/*z* 1046.5418), angiotensin I (*m*/*z* 1296.6848), substance P (*m*/*z* 1758.9326), ACTH clip 1–17 (*m*/*z* 2093.0862), ACTH18–39 (*m*/*z* 2465.1983) and somatostatin 28 (*m*/*z* 3147.4710). The MASCOT search engine was used to match the determined peptide masses to two customized databases: *Salmonella* Typhimurium from NCBI RefSeq (National Center for Biotechnology Information, U.S. National Library of Medicine, Bethesda, MD, USA), comprising 231,752 entries (Release 62); and *Salmonella* spp. from Swiss-Prot (Swiss Institute of Bioinformatics, Geneva, Switzerland; The EMBL Outstation—The European Bioinformatics Institute, Cambridge, UK), comprising 12,772 entries (Release 2013_11). The Max Planck Institute of Biochemistry, Martinsried, common contaminants collection (MPI) was included in both databases in order to avoid misleading matches in the presence of contaminant proteins. The search criteria adopted were: (i) proteolytic enzyme, trypsin/P; (ii) one missed cleavage allowed; (iii) fixed modifications, carbamidomethylation; (iv) variable modifications, methionine oxidation; and (v) a peptide tolerance error window up to 50 ppm. A match was considered significant when the probability of it being a random event was below the default significance threshold used (*p* < 0.05), *i.e.*, with a frequency less than 5%.

## 4. Conclusions

This study is a preliminary analysis of the proteomes of *S.* Typhimurium Se20 (phage type DT104) and SL1344 strains. It provides a physiological map and an overview of global protein expression of these strains under normal growth conditions, presented in the diverse context of *Salmonella* proteomics research. New stresses are continuously introduced to microbiological systems, contributing to the evolution of resistance mechanisms and spread of new resistance phenotypes. Discovering the physiological processes underlying these phenotypes is an important issue and microbial proteomics represents a powerful and accurate instrument for this purpose [11]. Additional work, such as comparative proteomics under antimicrobial stress conditions, will be developed to better understand the evolution of antimicrobial resistance in this pathogen.

## Supplementary Files

Supplementary File 1

## References

[1] Pacheco R., Correia S., Poeta P., Pinto L., Igrejas G., Annous B.A., Gurtler J.B. (2012). The role of proteomics in elucidating multiple antibiotic resistance in *Salmonella* and in novel antibacterial discovery. Salmonella—Distribution, Adaptation, Control Measures and Molecular Technologies.

[2] Su L.H., Wu T.L., Chiu C.H. (2012). Development of carbapenem resistance during therapy for non-typhoid *Salmonella* infection. Clin. Microbiol. Infect..

[3] Mather A.E., Reid S.W., Maskell D.J., Parkhill J., Fookes M.C., Harris S.R., Brown D.J., Coia J.E., Mulvey M.R., Gilmour M.W. (2013). Distinguishable epidemics of multidrug-resistant *Salmonella* Typhimurium DT104 in different hosts. Science.

[4] Marathe S.A., Kumar R., Ajitkumar P., Nagaraja V., Chakravortty D. (2013). Curcumin reduces the antimicrobial activity of ciprofloxacin against *Salmonella* Typhimurium and *Salmonella* Typhi. J. Antimicrob. Chemother..

[5] Bumann D. (2010). Pathogen proteomes during infection: A basis for infection research and novel control strategies. J. Proteomics.

[6] Humphrey S., Clark L.F., Humphrey T.J., Jepson M.A. (2011). Enhanced recovery of *Salmonella* Typhimurium DT104 from exposure to stress at low temperature. Microbiology.

[7] Fux C.A., Shirtliff M., Stoodley P., Costerton J.W. (2005). Can laboratory reference strains mirror “real-world” pathogenesis?. Trends Microbiol..

[8] Threlfall E.J. (2000). Epidemic *Salmonella* Typhimurium DT 104—A truly international multiresistant clone. J. Antimicrob. Chemother..

[9] Mendoza Mdel C., Herrero A., Rodicio M.R. (2009). Evolutionary engineering in *Salmonella*: Emergence of hybrid virulence-resistance plasmids in non-typhoid serotypes. Enferm. Infect. Microbiol. Clin..

[10] Giraud E., Baucheron S., Virlogeux-Payant I., Nishino K., Cloeckaert A. (2013). Effects of natural mutations in the *ramRA* locus on invasiveness of epidemic fluoroquinolone-resistant *Salmonella enterica* serovar Typhimurium isolates. J. Infect. Dis..

[11] Research Topic in Antimicrobials, Resistance and Chemotherapy. http://www.frontiersin.org/antimicrobials,_resistance_and_chemotherapy/researchtopics/proteomics_of_antimicrobial_re/1620.

[12] Fernandez-Reyes M., Rodriguez-Falcon M., Chiva C., Pachon J., Andreu D., Rivas L. (2009). The cost of resistance to colistin in *Acinetobacter baumannii*: A proteomic perspective. Proteomics.

[13] Becker D., Selbach M., Rollenhagen C., Ballmaier M., Meyer T.F., Mann M., Bumann D. (2006). Robust *Salmonella* metabolism limits possibilities for new antimicrobials. Nature.

[14] Lima T.B., Pinto M.F., Ribeiro S.M., de Lima L.A., Viana J.C., Gomes Junior N., Candido Ede S., Dias S.C., Franco O.L. (2013). Bacterial resistance mechanism: What proteomics can elucidate. FASEB J..

[15] Kleinert P., Kuster T., Arnold D., Jaeken J., Heizmann C.W., Troxler H. (2007). Effect of glycosylation on the protein pattern in 2-d-gel electrophoresis. Proteomics.

[16] Lee A.Y., Park S.G., Jang M., Cho S., Myung P.K., Kim Y.R., Rhee J.H., Lee D.H., Park B.C. (2006). Proteomic analysis of pathogenic bacterium *Vibrio vulnificus*. Proteomics.

[17] Vranakis I., Goniotakis I., Psaroulaki A., Sandalakis V., Tselentis Y., Gevaert K., Tsiotis G. (2014). Proteome studies of bacterial antibiotic resistance mechanisms. J. Proteomics.

[18] De Toro M., Rojo-Bezares B., Vinue L., Undabeitia E., Torres C., Saenz Y. (2010). *In vivo* selection of *aac(6')-Ib-cr* and mutations in the *gyrA* gene in a clinical *qnrS1*-positive *Salmonella enterica* serovar Typhimurium DT104B strain recovered after fluoroquinolone treatment. J. Antimicrob. Chemother..

[19] Hoiseth S.K., Stocker B.A. (1981). Aromatic-dependent *Salmonella* Typhimurium are non-virulent and effective as live vaccines. Nature.

[20] Wang Y., Huang K.Y., Huo Y. (2014). Proteomic comparison between *Salmonella* Typhimurium and *Salmonella* Typhi. J. Microbiol..

[21] Zhang Y., Nandakumar R., Bartelt-Hunt S.L., Snow D.D., Hodges L., Li X.  (2014). Quantitative proteomic analysis of the *Salmonella*-lettuce interaction. Microb. Biotechnol..

[22] Zhang L., Xiao D., Pang B., Zhang Q., Zhou H., Zhang L., Zhang J., Kan B. (2014). The core proteome and pan proteome of *Salmonella* Paratyphi A epidemic strains. PLoS One.

[23] Ansong C., Wu S., Meng D., Liu X., Brewer H.M., Deatherage Kaiser B.L., Nakayasu E.S., Cort J.R., Pevzner P., Smith R.D. (2013). Top-down proteomics reveals a unique protein S-thiolation switch in *Salmonella* Typhimurium in response to infection-like conditions. Proc. Natl. Acad. Sci. USA.

[24] Brown R.N., Sanford J.A., Park J.H., Deatherage B.L., Champion B.L., Smith R.D., Heffron F., Adkins J.N. (2012). A Comprehensive Subcellular Proteomic Survey of *Salmonella* Grown under Phagosome-Mimicking *versus* Standard Laboratory Conditions. Int. J. Proteomics.

[25] Condell O., Sheridan A., Power K.A., Bonilla-Santiago R., Sergeant K., Renaut J., Burgess C., Fanning S., Nally J.E. (2012). Comparative proteomic analysis of *Salmonella* tolerance to the biocide active agent triclosan. J. Proteomics.

[26] Cooper B., Chen R., Garrett W.M., Murphy C., Chang C., Tucker M.L., Bhagwat A.A. (2012). Proteomic pleiotropy of *OpgGH*, an operon necessary for efficient growth of *Salmonella enterica* serovar typhimurium under low-osmotic conditions. J. Proteome Res..

[27] Feng Y., Chien K.Y., Chen H.L., Chiu C.H. (2012). Pseudogene recoding revealed from proteomic analysis of *Salmonella* serovars. J. Proteome Res..

[28] Kang M.S., Kwon Y.K., Kim H.R., Oh J.Y., Kim M.J., An B.K., Shin E.G., Kwon J.H., Park C.K. (2012). Comparative proteome and transcriptome analyses of wild-type and live vaccine strains of *Salmonella enterica* serovar Gallinarum. Vaccine.

[29] Sun J.S., Hahn T.W. (2012). Comparative proteomic analysis of *Salmonella enterica* serovars Enteritidis, Typhimurium and Gallinarum. J. Vet. Med. Sci./Jpn. Soc. Vet. Sci..

[30] Ciavardelli D., Ammendola S., Ronci M., Consalvo A., Marzano V., Lipoma M., Sacchetta P., Federici G., di Ilio C., Battistoni A. (2011). Phenotypic profile linked to inhibition of the major Zn influx system in *Salmonella enterica*: Proteomics and ionomics investigations. Mol. Biosyst..

[31] Chen J., Wei D., Zhuang H., Qiao Y., Tang B., Zhang X., Wei J., Fang S., Chen G., Du P. (2011). Proteomic screening of anaerobically regulated promoters from Salmonella and its antitumor applications. Mol. Cell. Proteomics: MCP.

[32] Niemann G.S., Brown R.N., Gustin J.K., Stufkens A., Shaikh-Kidwai A.S., Li J., McDermott J.E., Brewer H.M., Schepmoes A., Smith R.D. (2011). Discovery of novel secreted virulence factors from *Salmonella enterica* serovar Typhimurium by proteomic analysis of culture supernatants. Infect. Immun..

[33] Paradela A., Mariscotti J.F., Navajas R., Ramos-Fernandez A., Albar J.P., Garcia-del Portillo F. (2011). Inverse regulation in the metabolic genes *pckA* and *metE* revealed by proteomic analysis of the Salmonella RcsCDB regulon. J. Proteome Res..

[34] Yu J.L., Guo L. (2011). Quantitative proteomic analysis of *Salmonella enterica* serovar Typhimurium under PhoP/PhoQ activation conditions. J. Proteome Res..

[35] Charles R.C., Sheikh A., Krastins B., Harris J.B., Bhuiyan M.S., LaRocque R.C., Logvinenko T., Sarracino D.A., Kudva I.T., Eisenstein J. (2010). Characterization of anti-*Salmonella enterica* serotype Typhi antibody responses in bacteremic Bangladeshi patients by an immunoaffinity proteomics-based technology. Clin. Vaccine Immunol.: CVI.

[36] Beraud M., Kolb A., Monteil V., D’Alayer J., Norel F. (2010). A proteomic analysis reveals differential regulation of the σ^S^-dependent *yciGFE*(*katN*) locus by YncC and H-NS in *Salmonella* and *Escherichia coli* K-12. Mol. Cell. Proteomics: MCP.

[37] Calhoun L.N., Liyanage R., Lay J.O., Kwon Y.M. (2010). Proteomic analysis of *Salmonella enterica* serovar Enteritidis following propionate adaptation. BMC Microbiol..

[38] Kim K., Yang E., Vu G.P., Gong H., Su J., Liu F., Lu S. (2010). Mass spectrometry-based quantitative proteomic analysis of *Salmonella enterica* serovar Enteritidis protein expression upon exposure to hydrogen peroxide. BMC Microbiol..

[39] Pinto L., Poeta P., Vieira S., Caleja C., Radhouani H., Carvalho C., Vieira-Pinto M., Themudo P., Torres C., Vitorino R. (2010). Genomic and proteomic evaluation of antibiotic resistance in *Salmonella* strains. J. Proteomics.

[40] Chooneea D., Karlsson R., Encheva V., Arnold C., Appleton H., Shah H. (2010). Elucidation of the outer membrane proteome of *Salmonella enterica* serovar Typhimurium utilising a lipid-based protein immobilization technique. BMC Microbiol..

[41] Di Pasqua R., Mamone G., Ferranti P., Ercolini D., Mauriello G. (2010). Changes in the proteome of *Salmonella enterica* serovar Thompson as stress adaptation to sublethal concentrations of thymol. Proteomics.

[42] Charles R.C., Harris J.B., Chase M.R., Lebrun L.M., Sheikh A., LaRocque R.C., Logvinenko T., Rollins S.M., Tarique A., Hohmann E.L. (2009). Comparative proteomic analysis of the PhoP regulon in *Salmonella enterica* serovar Typhi *versus* Typhimurium. PLoS One.

[43] Encheva V., Shah H.N., Gharbia S.E. (2009). Proteomic analysis of the adaptive response of *Salmonella enterica* serovar Typhimurium to growth under anaerobic conditions. Microbiology.

[44] Kim S.R., Kim H.T., Park H.J., Kim S., Choi H.J., Hwang G.S., Yi J.H., Ryu do H., Kim K.H. (2009). Fatty acid profiling and proteomic analysis of *Salmonella enterica* serotype Typhimurium inactivated with supercritical carbon dioxide. Int. J. Food Microbiol..

[45] Osman K.M., Ali M.M., Radwan M.I., Kim H.K., Han J. (2009). Comparative proteomic analysis on *Salmonella* Gallinarum and *Salmonella* Enteritidis exploring proteins that may incorporate host adaptation in poultry. J. Proteomics.

[46] Shi L., Chowdhury S.M., Smallwood H.S., Yoon H., Mottaz-Brewer H.M., Norbeck A.D., McDermott J.E., Clauss T.R., Heffron F., Smith R.D. (2009). Proteomic investigation of the time course responses of RAW 264.7 macrophages to infection with *Salmonella enterica*. Infect. Immun..

[47] Shi L., Ansong C., Smallwood H., Rommereim L., McDermott J.E., Brewer H.M., Norbeck A.D., Taylor R.C., Gustin J.K., Heffron F. (2009). Proteome of *Salmonella enterica* serotype Typhimurium grown in a Low Mg/pH medium. J. Proteomics Bioinform..

[48] Kint G., Sonck K.A., Schoofs G., de Coster D., Vanderleyden J., de Keersmaecker S.C. (2009). 2D proteome analysis initiates new insights on the *Salmonella* Typhimurium LuxS protein. BMC Microbiol..

[49] Sonck K.A., Kint G., Schoofs G., Vander Wauven C., Vanderleyden J., de Keersmaecker S.C. (2009). The proteome of *Salmonella* Typhimurium grown under *in vivo*-mimicking conditions. Proteomics.

[50] Webber M.A., Coldham N.G., Woodward M.J., Piddock L.J. (2008). Proteomic analysis of triclosan resistance in *Salmonella enterica* serovar Typhimurium. J. Antimicrob. Chemother..

[51] Singh R., Shasany A.K., Aggarwal A., Sinha S., Sisodia B.S., Khanuja S.P., Misra R. (2007). Low molecular weight proteins of outer membrane of *Salmonella* Typhimurium are immunogenic in *Salmonella* induced reactive arthritis revealed by proteomics. Clin. Exp. Immunol..

[52] Hu W.S., Lin Y.H., Shih C.C. (2007). A proteomic approach to study *Salmonella enterica* serovar Typhimurium putative transporter YjeH associated with ceftriaxone resistance. Biochem. Biophys. Res. Commun..

[53] Nunn B.L., Shaffer S.A., Scherl A., Gallis B., Wu M., Miller S.I., Goodlett D.R. (2006). Comparison of a *Salmonella* Typhimurium proteome defined by shotgun proteomics directly on an LTQ-FT and by proteome pre-fractionation on an LCQ-DUO. Brief. Funct. Genomics Proteomics.

[54] Shi L., Adkins J.N., Coleman J.R., Schepmoes A.A., Dohnkova A., Mottaz H.M., Norbeck A.D., Purvine S.O., Manes N.P., Smallwood H.S. (2006). Proteomic analysis of *Salmonella enterica* serovar Typhimurium isolated from RAW 264.7 macrophages: Identification of a novel protein that contributes to the replication of serovar Typhimurium inside macrophages. J. Biol. Chem..

[55] Coldham N.G., Randall L.P., Piddock L.J., Woodward M.J. (2006). Effect of fluoroquinolone exposure on the proteome of *Salmonella enterica* serovar Typhimurium. J. Antimicrob. Chemother..

[56] Adkins J.N., Mottaz H.M., Norbeck A.D., Gustin J.K., Rue J., Clauss T.R., Purvine S.O., Rodland K.D., Heffron F., Smith R.D. (2006). Analysis of the *Salmonella* Typhimurium proteome through environmental response toward infectious conditions. Mol. Cell. Proteomics: MCP.

[57] Encheva V., Wait R., Gharbia S.E., Begum S., Shah H.N. (2005). Proteome analysis of serovars Typhimurium and Pullorum of *Salmonella enterica* subspecies I. BMC Microbiol..

[58] Agudo D., Mendoza M.T., Castanares C., Nombela C., Rotger R. (2004). A proteomic approach to study *Salmonella* typhi periplasmic proteins altered by a lack of the DsbA thiol: Disulfide isomerase. Proteomics.

[59] Coldham N.G., Woodward M.J. (2004). Characterization of the *Salmonella* Typhimurium proteome by semi-automated two dimensional HPLC-mass spectrometry: Detection of proteins implicated in multiple antibiotic resistance. J. Proteome Res..

[60] Yoon H., Lim S., Heu S., Choi S., Ryu S. (2003). Proteome analysis of *Salmonella enterica* serovar Typhimurium *fis* mutant. FEMS Microbiol. Lett..

[61] Deiwick J., Rappl C., Stender S., Jungblut P.R., Hensel M. (2002). Proteomic approaches to *Salmonella* Pathogenicity Island 2 encoded proteins and the SsrAB regulon. Proteomics.

[62] Adams P., Fowler R., Kinsella N., Howell G., Farris M., Coote P., O’Connor C.D. (2001). Proteomic detection of PhoPQ- and acid-mediated repression of *Salmonella* motility. Proteomics.

[63] O’Connor C.D., Farris M., Fowler R., Qi S.Y. (1997). The proteome of *Salmonella enterica* serovar Typhimurium: Current progress on its determination and some applications. Electrophoresis.

[64] Qi S.Y., Moir A., O’Connor C.D. (1996). Proteome of *Salmonella* Typhimurium SL1344: Identification of novel abundant cell envelope proteins and assignment to a two-dimensional reference map. J. Bacteriol..

[65] Hogg G., Dimovski K., Hiley L., Holt K.E. (2013). Draft Genome Sequences for Ten *Salmonella enterica* Serovar Typhimurium Phage Type 135 Variants. Genome Announc..

[66] De Toro M., Rodriguez I., Rojo-Bezares B., Helmuth R., Torres C., Guerra B., Saenz Y. (2013). pMdT1, a small ColE1-like plasmid mobilizing a new variant of the *aac(6')-Ib-cr* gene in *Salmonella enterica* serovar Typhimurium. J. Antimicrob. Chemother..

[67] Rankin J.D., Taylor R.J. (1966). The estimation of doses of *Salmonella* Typhimurium suitable for the experimental production of disease in calves. Vet. Rec..

[68] Stecher B., Robbiani R., Walker A.W., Westendorf A.M., Barthel M., Kremer M., Chaffron S., Macpherson A.J., Buer J., Parkhill J. (2007). *Salmonella enterica* serovar Typhimurium exploits inflammation to compete with the intestinal microbiota. PLoS Biol..

[69] Supek F., Bosnjak M., Skunca N., Smuc T. (2011). REVIGO summarizes and visualizes long lists of gene ontology terms. PLoS One.

[70] Poirel L., Cattoir V., Nordmann P. (2012). Plasmid-Mediated Quinolone Resistance; Interactions between Human, Animal, and Environmental Ecologies. Front. Microbiol..

[71] Cattoir V., Nordmann P. (2009). Plasmid-mediated quinolone resistance in gram-negative bacterial species: An update. Curr. Med. Chem..

[72] Marchler-Bauer A., Zheng C., Chitsaz F., Derbyshire M.K., Geer L.Y., Geer R.C., Gonzales N.R., Gwadz M., Hurwitz D.I., Lanczycki C.J. (2013). CDD: Conserved domains and protein three-dimensional structure. Nucleic Acids Res..

[73] Righetti P.G. (2006). Real and imaginary artefacts in proteome analysis via two-dimensional maps. J. Chromatogr. B, Anal. Technol. Biomed. Life Sci..

[74] Skold O. (2000). Sulfonamide resistance: Mechanisms and trends. Drug Resist. Updates.

[75] Kroger C., Dillon S.C., Cameron A.D., Papenfort K., Sivasankaran S.K., Hokamp K., Chao Y., Sittka A., Hebrard M., Handler K. (2012). The transcriptional landscape and small RNAs of *Salmonella enterica* serovar Typhimurium. Proc. Natl. Acad. Sci. USA.

[76] Lal A., Vela J., Yee J., Yuan E. (2012). Rescuing the *ompA* Deletion Mutant *Escherichia coli* JW0940 by Reintroducing *ompA* in the TOPO Cloning Vector pBAD. J. Exp. Microbiol. Immunol..

[77] Kleinschmidt J.H. (2006). Folding kinetics of the outer membrane proteins OmpA and FomA into phospholipid bilayers. Chem. Phys. Lipids.

[78] Mahendran K.R., Kreir M., Weingart H., Fertig N., Winterhalter M. (2010). Permeation of antibiotics through *Escherichia coli* OmpF and OmpC porins: Screening for influx on a single-molecule level. J. Biomol. Screen..

[79] Paterson G.K., Northen H., Cone D.B., Willers C., Peters S.E., Maskell D.J. (2009). Deletion of *tolA* in *Salmonella* Typhimurium generates an attenuated strain with vaccine potential. Microbiology.

[80] Nirudodhi S., Parsonage D., Karplus P.A., Poole L.B., Maier C.S. (2011). Conformational studies of the robust 2-Cys peroxiredoxin *Salmonella* Typhimurium AhpC by solution phase hydrogen/deuterium (H/D) exchange monitored by electrospray ionization mass spectrometry. Int. J. Mass Spectrom..

[81] Guerrero P., Collao B., Alvarez R., Salinas H., Morales E.H., Calderon I.L., Saavedra C.P., Gil F. (2013). Salmonella enterica serovar Typhimurium BaeSR two-component system positively regulates *sodA* in response to ciprofloxacin. Microbiology.

[82] Bakshi C.S., Malik M., Regan K., Melendez J.A., Metzger D.W., Pavlov V.M., Sellati T.J. (2006). Superoxide dismutase B gene (*sodB*)-deficient mutants of *Francisella tularensis* demonstrate hypersensitivity to oxidative stress and attenuated virulence. J. Bacteriol..

[83] Troxell B., Fink R.C., Porwollik S., McClelland M., Hassan H.M. (2011). The Fur regulon in anaerobically grown *Salmonella enterica* sv. Typhimurium: Identification of new Fur targets. BMC Microbiol..

[84] Yang X., Thornburg T., Suo Z., Jun S., Robison A., Li J., Lim T., Cao L., Hoyt T., Avci R. (2012). Flagella overexpression attenuates *Salmonella* pathogenesis. PLoS One.

[85] Garsin D.A. (2010). Ethanolamine utilization in bacterial pathogens: Roles and regulation. Nat. Rev. Microbiol..

[86] Thiennimitr P., Winter S.E., Winter M.G., Xavier M.N., Tolstikov V., Huseby D.L., Sterzenbach T., Tsolis R.M., Roth J.R., Baumler A.J. (2011). Intestinal inflammation allows *Salmonella* to use ethanolamine to compete with the microbiota. Proc. Natl. Acad. Sci. USA.

[87] Rittmann D., Sorger-Herrmann U., Wendisch V.F. (2005). Phosphate starvation-inducible gene *ushA* encodes a 5' nucleotidase required for growth of *Corynebacterium glutamicum* on media with nucleotides as the phosphorus source. Appl. Environ. Microbiol..

[88] Choi Y., Choi J., Groisman E.A., Kang D.H., Shin D., Ryu S. (2012). Expression of STM4467-encoded arginine deiminase controlled by the STM4463 regulator contributes to *Salmonella enterica* serovar Typhimurium virulence. Infect. Immun..

[89] Cohen-Ben-Lulu G.N., Francis N.R., Shimoni E., Noy D., Davidov Y., Prasad K., Sagi Y., Cecchini G., Johnstone R.M., Eisenbach M. (2008). The bacterial flagellar switch complex is getting more complex. EMBO J..

[90] Liechti G., Goldberg J.B. (2012). *Helicobacter pylori* relies primarily on the purine salvage pathway for purine nucleotide biosynthesis. J. Bacteriol..

[91] Suvarnapunya A.E., Lagasse H.A., Stein M.A. (2003). The role of DNA base excision repair in the pathogenesis of *Salmonella enterica* serovar Typhimurium. Mol. Microbiol..

[92] Turner A.K., Barber L.Z., Wigley P., Muhammad S., Jones M.A., Lovell M.A., Hulme S., Barrow P.A. (2003). Contribution of proton-translocating proteins to the virulence of *Salmonella enterica* serovars Typhimurium, Gallinarum, and Dublin in chickens and mice. Infect. Immun..

[93] Maier R.J., Olczak A., Maier S., Soni S., Gunn J. (2004). Respiratory hydrogen use by *Salmonella enterica* serovar Typhimurium is essential for virulence. Infect. Immun..

[94] Paterson G.K., Cone D.B., Peters S.E., Maskell D.J. (2009). The enzyme phosphoglucomutase (Pgm) is required by *Salmonella enterica* serovar Typhimurium for O-antigen production, resistance to antimicrobial peptides and *in vivo* fitness. Microbiology.

[95] Calhoun L.N., Kim J.N., Ren Y., Song J.J., Kwon Y.M. (2011). The DNA-binding protein dps functions as a global regulator in starved *Salmonella enterica* serovar enteritidis during starvation. Int. J. Microbiol. Res..

[96] Raffatellu M., Wilson R.P., Chessa D., Andrews-Polymenis H., Tran Q.T., Lawhon S., Khare S., Adams L.G., Baumler A.J. (2005). SipA, SopA, SopB, SopD, and SopE2 contribute to *Salmonella enterica* serotype Typhimurium invasion of epithelial cells. Infect. Immun..

[97] Slattery A., Victorsen A.H., Brown A., Hillman K., Phillips G.J. (2013). Isolation of highly persistent mutants of *Salmonella enterica* serovar Typhimurium reveals a new toxin-antitoxin module. J. Bacteriol..

[98] Bradford M.M. (1976). A rapid and sensitive method for the quantitation of microgram quantities of protein utilizing the principle of protein-dye binding. Anal. Biochem..

[99] O’Farrell P.H. (1975). High resolution two-dimensional electrophoresis of proteins. J. Biol. Chem..

[100] Görg A., Klaus A., Lück C., Weiland F., Weiss W. (2007). Two-Dimensional Electrophoresis with Immobilized pH Gradients for Proteome Analysis: A laboratory manual.

[101] Gorg A., Obermaier C., Boguth G., Harder A., Scheibe B., Wildgruber R., Weiss W. (2000). The current state of two-dimensional electrophoresis with immobilized pH gradients. Electrophoresis.

[102] Laemmli U.K. (1970). Cleavage of structural proteins during the assembly of the head of bacteriophage T4. Nature.

[103] Gorg A., Weiss W., Dunn M.J. (2004). Current two-dimensional electrophoresis technology for proteomics. Proteomics.

