# Supplementary material for: Complete Proteome of a Quinolone-Resistant Salmonella Typhimurium Phage Type DT104B Clinical Strain
Source: Int J Mol Sci. 2014 Aug 15;15(8):14191–219. doi: 10.3390/ijms150814191 (PMC4159846; doi:10.3390/ijms150814191)
Supplement: Supplementary File 1 [file ijms-15-14191-s001.pdf]

Supplementary Information

Table S1. *Salmonella* Typhimurium Se20 (phage type DT104B) proteins identified by MALDI-TOF MS.

Spot		GI Number	UniProt AC	Protein Name	Gene Names	Protein Length	Protein M_W (Da)	pI Value	Mascot Score	MS Coverage	Biological Process
1	*	525839731	S5HK16	molecular chaperone GroEL	groEL	548	57,421	4.7	350	71	protein folding, protein refolding
2	*	549591826	U6W8M2	elongation factor Tu, partial	tuf	388	42,767	5.1	358	77	GTP catabolic process, translational elongation
3	*	525838467	S5H702	glyceraldehyde-3-phosphate dehydrogenase		331	35,735	6.4	310	75	glucose metabolic process, oxidation-reduction process
4	*	383495039	H8M4U4	30S ribosomal protein S2	rpsB	236	26,310	6.7	213	61	translation
5		525839734	S5HHN2	aspartate ammonia lyase	aspA	478	52,880	5	252	52	tricarboxylic acid cycle, aspartate metabolic process
6	*	545007466	Q8Z7S0	outer membrane protein A	ompA	350	37,606	5.5	209	52	conjugation, transmembrane ion transport
7	*	525837154	S5HA44	enolase	eno	432	45,627	5.1	312	74	glycolysis
8	*	383497733	H8M1H4	phosphoglycerate kinase	pgk	382	40,719	4.9	233	57	glycolysis, phosphorylation
9	*	525839509	S5HGY3	molecular chaperone DnaK	dnaK	638	69,273	4.7	358	52	response to stress, protein folding
10		529292027	not mapped	30S ribosomal protein S1, partial			44,495	4.8	142	50	
11	*	545006954	T2K3Y0	heat shock protein HtpG	htpG	632	72,364	5.1	312	53	protein folding, response to stress
12	*	525840415	S5H8L9	elongation factor G	fusA	704	77722	5	365	57	GTP catabolic process, translational elongation
13	*	525840415	S5H8L9	elongation factor G	fusA	704	77,722	5	421	64	GTP catabolic process, translational elongation
14	*	525837344	S5HAQ1	protein disaggregation chaperone		857	95,549	5.2	431	60	response to heat, protein processing

Table S1. *Cont.*

Spot		GI Number	UniProt AC	Protein Name	Gene Names	Protein Length	Protein M_W (Da)	pI Value	Mascot Score	MS Coverage	Biological Process
15		525839720	S5H6L3	fumarate reductase flavoprotein subunit		596	66,021	5.9	292	49	electron transport chain, oxidation-reduction process
16		525839720	S5H6L3	fumarate reductase flavoprotein subunit		596	66,021	5.9	314	49	electron transport chain, oxidation-reduction process
17	*	525838467	S5H702	glyceraldehyde-3-phosphate dehydrogenase		331	35,735	6.4	296	74	glucose metabolic process, oxidation-reduction process
18	*	525840042	S5HBA0	transcription termination factor Rho	rho	419	47,021	6.9	162	42	ATP catabolic process, DNA-templated transcription, termination
19		525836798	S5H107	acetyl-CoA carboxylase biotin carboxylase subunit		449	49,630	6.6	252	59	metabolic process
20	*	525838467	S5H702	glyceraldehyde-3-phosphate dehydrogenase		331	35,735	6.4	197	69	glucose metabolic process, oxidation-reduction process
21	*	525838467	S5H702	glyceraldehyde-3-phosphate dehydrogenase		331	35,735	6.4	248	64	glucose metabolic process, oxidation-reduction process
22		525837064	S5H9U4	fructose-bisphosphate aldolase		359	39,360	5.6	116	35	glycolysis, carbohydrate metabolic process
23	*	525839101	S5HI58	trigger factor	tig	432	48,037	4.7	398	65	protein peptidyl-prolyl isomerization, cell cycle, protein transport
24	*	525837296	S5HD40	flagellin		506	52,504	4.6	254	52	ciliary or bacterial-type flagellar motility
25	*	525840077	S5HBD9	F_0F_1 ATP synthase subunit beta	atpD	460	50,309	4.8	322	69	plasma membrane ATP synthesis coupled proton transport, ATP hydrolysis coupled proton transport

Table S1. *Cont.*

Spot		GI Number	UniProt AC	Protein Name	Gene Names	Protein Length	Protein M_W (Da)	pI Value	Mascot Score	MS Coverage	Biological Process
26	*	525840441	S5HLY6	DNA-directed RNA polymerase subunit alpha	rpoA	329	36,717	4.8	260	61	transcription, DNA-templated
27	*	525839514	S5HGY9	transaldolase	tal	317	35,320	5	164	48	carbohydrate metabolic process, pentose-phosphate shunt
28	*	525839322	S5H9F3	endo-1,4-D-glucanase	tsf	283	30,453	5	215	51	translational elongation
29	*	525839927	S5HAZ7	6-phosphofructokinase	pfkA	320	35,235	5.5	135	50	fructose 6-phosphate metabolic process, glycolysis, carbohydrate phosphorylation
30	*	525838135	S5H284	enoyl-ACP reductase		262	27,971	5.5	116	41	fatty acid biosynthetic process, oxidation-reduction process
31	*	532641272	T5JVY4	phosphoglyceromutase, partial	gpmA	243	27,736	5.4	146	44	glycolysis
32	*	525839546	S5HJF3	purine nucleoside phosphorylase	deoD	239	26,189	5.5	206	76	purine nucleoside metabolic process
33		525839140	S5H512	peroxiredoxin		200	22,417	5.1	190	68	oxidation-reduction process
34	*	525839858	S5H9S7	DNA-directed RNA polymerase subunit beta	rpoB	1342	150,876	5	445	38	transcription, DNA-templated
35	*	525840415	S5H8L9	elongation factor G	fusA	704	77,722	5	256	44	GTP catabolic process, translational elongation
36		525838875	S5H4D5	2-oxoglutarate dehydrogenase E1		933	105,328	6.1	195	25	tricarboxylic acid cycle, oxidation-reduction process
37		525838094	S5HCW3	acetaldehyde dehydrogenase		892	96,668	6.2	187	34	alcohol metabolic process, carbon utilization, oxidation-reduction process

Table S1. *Cont.*

Spot		GI Number	UniProt AC	Protein Name	Gene Names	Protein Length	Protein M_W (Da)	pI Value	Mascot Score	MS Coverage	Biological Process
39		525839098	S5H7L3	peptidase	lon	784	87,698	6	213	37	response to stress, proteolysis, misfolded or incompletely synthesized protein catabolic process
40		532640808	K8W3Y2	dimethyl sulfoxide reductase subunit A		740	83,389	5.8	421	53	oxidation-reduction process
41	*	525840102	S5HBG1	DNA gyrase subunit B	gyrB	804	90,125	5.7	248	36	DNA topological change, ATP catabolic process
42		525838441	S5HGB6	hydroperoxidase II	katE	750	83,802	5.7	287	33	hydrogen peroxide catabolic process, oxidation-reduction process
43		525839734	S5HHN2	aspartate ammonia-lyase	aspA	478	52,880	5	170	46	tricarboxylic acid cycle, aspartate metabolic process
44	*	525840371	S5HLS6	phosphoenolpyruvate carboxykinase	pckA	539	59,895	5.6	376	71	gluconeogenesis, phosphorylation
45	*	525840075	S5H7K0	F_0F_1 ATP synthase subunit alpha	atpA	513	55,307	5.6	320	57	ATP hydrolysis coupled proton transport, plasma membrane ATP synthesis coupled proton transport
46	*	525838719	S5HEM6	seryl-tRNA synthetase	serS	430	48,835	5.3	145	42	seryl-tRNA aminoacylation, selenocysteinyl-tRNA(Sec) biosynthetic process
47		525839380	S5H5Q6	dihydrolipoamide dehydrogenase		474	50,893	5.8	279	55	cell redox homeostasis
48	*	525836878	S5H169	translation initiation factor IF-2	infB	892	97,514	5.8	214	27	GTP catabolic process, translational initiation

Table S1. *Cont.*

Spot		GI Number	UniProt AC	Protein Name	Gene Names	Protein Length	Protein M_W (Da)	pI Value	Mascot Score	MS Coverage	Biological Process
49		525839377	S5H9J9	bifunctional aconitate hydratase 2/2-methylisocitrate dehydratase		865	94,040	5.1	208	25	tricarboxylic acid cycle
50	*	525840415	S5H8L9	elongation factor G	fusA	704	77,722	5	108	21	GTP catabolic process, translational elongation
51		525839382	S5H9K2	pyruvate dehydrogenase	aceE	887	99,802	5.4	267	30	oxidation-reduction process
52	*	525839315	S5H5L1	outer membrane protein assembly factor YaeT	bamA	804	89,585	4.8	199	37	Gram-negative-bacterium-type cell outer membrane assembly, protein insertion into membrane
53	*	81521548	Q8ZLT3	polyribonucleotide nucleotidyltransferase	pnp	711	77,048	4.9	326	39	RNA phosphodiester bond hydrolysis, exonucleolytic, mRNA catabolic process
54		525837570	S5H0M0	phosphate acetyltransferase		714	77,572	5.2	125	30	acetyl-CoA biosynthetic process
55		525837456	S5HDG7	malic enzyme		759	82,726	5.4	224	31	malate metabolic process, oxidation-reduction process
56	*	525837570	S5H0M0	phosphate acetyltransferase		714	77,572	5.2	223	44	acetyl-CoA biosynthetic process
57	*	525841024	S5HLB3	heat shock protein 90	htpG	624	71,442	5	109	29	protein folding, response to stress
58		525838733	S5H6L9	ATP-dependent Clp protease ATP-binding protein	clpA	758	84,156	5.8	214	29	ATP catabolic process, proteolysis
59		525839734	S5HHN2	aspartate ammonia-lyase	aspA	478	52,880	5	184	38	tricarboxylic acid cycle, aspartate metabolic process

Table S1. *Cont.*

Spot		GI Number	UniProt AC	Protein Name	Gene Names	Protein Length	Protein M_W (Da)	pI Value	Mascot Score	MS Coverage	Biological Process
60	*	525837491	S5HDK7	phosphoenolpyruvate-protein phosphotransferase		575	63,557	4.6	138	31	phosphoenolpyruvate-dependent sugar phosphotransferase system, phosphorylation
61	*	545007761	T2K502	pyruvate kinase	pykF	452	48,907	5.4	168	45	glycolysis, phosphorylation
62	*	525837987	S5H5K3	pyruvate kinase		480	51,583	6.1	155	33	glycolysis, phosphorylation
63		525836996	S5HC51	hydrogenase 2 large subunit		567	62,912	5.8	231	44	oxidation-reduction process
64	*	525838467	S5H702	glyceraldehyde-3-phosphate dehydrogenase		331	35,735	6.4	165	39	glucose metabolic process, oxidation-reduction process
65		525838024	S5HCN7	PTS mannose transporter subunit IIAB		322	34,965	5.8	262	64	phosphoenolpyruvate-dependent sugar phosphotransferase system
66		525838024	S5HCN7	PTS mannose transporter subunit IIAB		322	34,965	5.8	240	63	phosphoenolpyruvate-dependent sugar phosphotransferase system
67	*	525836819	S5H969	malate dehydrogenase	mdh	312	32,626	6	157	47	malate metabolic process, carbohydrate metabolic process, tricarboxylic acid cycle, oxidation-reduction process
68	*	525838611	S5HGT7	DNA-binding protein	cbpA	306	34,672	6.6	103	37	protein folding
69		525840408	S5HBH2	peptidyl-prolyl *cis-trans* isomerase		272	28,927	9	136	53	protein peptidyl-prolyl isomerization
70	*	525838914	S5HF78	glucosamine-6-phosphate deaminase	nagB	266	29,784	6.4	128	47	*N*-acetylglucosamine metabolic process
71	*	525841256	P0A2C6	D-ribose-binding periplasmic protein	RbsB	296	30,943	9.2	178	43	chemotaxis, carbohydrate transport
72	*	525840420	S5H8M3	50S ribosomal protein L4	rplD	201	22,073	10.2	92	39	translation

Table S1. *Cont.*

Spot		GI Number	UniProt AC	Protein Name	Gene Names	Protein Length	Protein M_W (Da)	pI Value	Mascot Score	MS Coverage	Biological Process
73		525838341	S5HG46	glutathionine *S*-transferase		201	22,543	6.1	143	59	metabolic process
74	*	525839914	S5HI71	triosephosphate isomerase	tpiA	255	27,071	5.6	168	52	gluconeogenesis, glycolysis, pentose-phosphate shunt
75		410655108	K4I0G8	aminoglycoside 6'-*N*-acetyltransferase type Ib-cr, AAC(6')-Ib-cr4	aac(6') -Ib-cr4	225	25,031	5.2	144	46	metabolic process
76	*	414022212	K8SG73	DNA-binding transcriptional regulator PhoP		223	25,486	5.2	213	62	phosphorelay signal transduction system, transcription, DNA-templated
77	*	525838356	P0A2F5	superoxide dismutase [Fe]	sodB	193	21,352	5.5	118	55	oxidation-reduction process, superoxide metabolic process
78		549725641	U4MED2	transketolase	tktA	648	70,745	5.2	164	20	metabolic process
79	*	525840248	S5HB18	glycyl-tRNA synthetase	glyS	689	76,576	5.4	252	44	arginyl-tRNA aminoacylation, glycyl-tRNA aminoacylation
80	*	525839673	S5H982	3'-nucleotidase	cpdB	647	70,587	5.6	151	33	dephosphorylation, nucleotide catabolic process
81	*	525838912	S5H878	glutaminyl-tRNA synthetase	glnS	555	64,068	5.6	220	36	glutamyl-tRNA aminoacylation, glutaminyl-tRNA aminoacylation
82	*	525837153	S5H1Y1	CTP synthetase	pyrG	545	60,540	5.6	192	45	*de novo* CTP biosynthetic process, glutamine metabolic process
83		525837616	S5HDZ5	*sn*-glycerol-3-phosphate dehydrogenase subunit A	glpA	542	59,819	6.2	363	61	glycerol-3-phosphate metabolic process, polyol catabolic process, oxidation-reduction process
84		525838742	S5H7T6	pyruvate dehydrogenase		572	62,214	6.3	117	26	metabolic process

Table S1. *Cont.*

Spot		GI Number	UniProt AC	Protein Name	Gene Names	Protein Length	Protein M_W (Da)	pI Value	Mascot Score	MS Coverage	Biological Process
85	*	54041937	P67557	glucans biosynthesis protein G Precursor	mdoG	511	57,808	8.8	212	42	glucan biosynthetic process
87	*	525839748	S5H9G5	fumarate hydratase		548	60,523	5.9	137	31	generation of precursor metabolites and energy
88	*	383498724	H8M0S2	pyridine nucleotide-disulfide oxidoreductase	sthA	444	49,878	6.2	127	33	NADP metabolic process, cell redox homeostasis, hydrogen ion transmembrane transport
89		525838959	S5HFC4	citrate lyase subunit alpha		509	55,103	6.3	117	32	acetyl-CoA metabolic process
90		525836996	S5HC51	hydrogenase 2 large subunit		567	62,912	5.8	184	38	oxidation-reduction process
91		525836996	S5HC51	hydrogenase 2 large subunit		567	62,912	5.8	114	24	oxidation-reduction process
92	*	525839990	S5H7C8	proline dipeptidase	pepQ	443	50,309	5.8	178	42	proteolysis
93		525837137	S5H337	L-serine dehydratase		455	49,449	5.8	121	21	gluconeogenesis
94	*	525837388	S5H2M8	serine hydroxymethyltransferase	glyA	417	45,597	6	180	38	cellular amino acid biosynthetic process, methylation, one-carbon metabolic process
95		525836915	S5GYP6	L-serine dehydratase		454	48,957	6	163	38	gluconeogenesis
96	*	383498489	H8M229	UDP-*N*-acetylglucosamine pyrophosphorylase	glmU	451	48,936	6.3	97	30	UDP-*N*-acetylglucosamine biosynthetic process, regulation of cell shape, lipid A biosynthetic process
97	*	383498339	H8LYI4	2,3-bisphosphoglycerate-independent phosphoglycerate mutase	gpmI	523	57,355	4.9	142	47	glycolysis

Table S1. *Cont.*

Spot		GI Number	UniProt AC	Protein Name	Gene Names	Protein Length	Protein M_W (Da)	pI Value	Mascot Score	MS Coverage	Biological Process
98	*	525837296	S5HD40	flagellin		506	52,504	4.6	163	40	ciliary or bacterial-type flagellar motility
99	*	383497152	H8LX56	ethanolamine ammonia-lyase heavy subunit	eutB	447	49,303	4.6	245	50	cellular amino acid metabolic process
100	*	116489820	A0FLL2	phase I flagellin middle domain variant C12	fliC	386	40,713	4.6	199	66	ciliary or bacterial-type flagellar motility
101		378443762	C9X823	aminoacyl-histidine dipeptidase precursor		485	52,693	5	97	31	proteolysis
102		525839734	S5HHN2	aspartate ammonia-lyase	aspA	478	52,880	5	175	46	tricarboxylic acid cycle, aspartate metabolic process
103		525839734	S5HHN2	aspartate ammonia-lyase	aspA	478	52,880	5	134	47	tricarboxylic acid cycle, aspartate metabolic process
104	*	525837775	S5H1A5	lipopolysaccharide biosynthesis protein		437	48,530	5.1	236	41	metabolic process
105	*	525839909	S5HI67	glycerol kinase	glpK	502	56,301	5.3	327	53	glycerol-3-phosphate metabolic process, phosphorylation, carbohydrate metabolic process
106	*	525839904	S5HI62	ATP-dependent protease	hslU	443	49,751	5.3	199	47	ATP catabolic process, proteolysis, response to stress, protein unfolding
107	*	525839547	S5HA11	phosphopentomutase	deoB	407	44,558	5	213	45	cellular metabolic compound salvage, 5-phosphoribose 1-diphosphate biosynthetic process, deoxyribonucleotide catabolic process

Table S1. *Cont.*

Spot		GI Number	UniProt AC	Protein Name	Gene Names	Protein Length	Protein M_W (Da)	pI Value	Mascot Score	MS Coverage	Biological Process
108		525838502	S5H738	isocitrate dehydrogenase		416	46,101	5	272	53	glyoxylate cycle, tricarboxylic acid cycle, oxidation-reduction process
109	*	525837048	S5H1M6	*S*-adenosylmethionine synthetase	metK	384	42,154	5	111	22	*S*-adenosylmethionine biosynthetic process, one-carbon metabolic process
110		380009294	H9CZL8	heat shock protein GroEL	groL	548	57,463	4.7	60	15	response to stress, protein folding, protein refolding
111		525838066	S5HF84	GTP-binding protein YchF	ychF	363	39,919	4.7	117	33	ATP catabolic process
112	*	549591826	U6W8M2	elongation factor Tu, partial	tuf	388	42,767	5.1	200	52	GTP catabolic process, translational elongation
113	*	549591826	U6W8M2	elongation factor Tu, partial	tuf	388	42,767	5.1	251	58	GTP catabolic process, translational elongation
114	*	549591826	U6W8M2	elongation factor Tu, partial	tuf	388	42,767	5.1	197	53	GTP catabolic process, translational elongation
115	*	525837008	S5H1I2	mannonate dehydratase	uxuA	394	45,080	5.6	85	29	glucuronate catabolic process
116	*	525837399	S5HAU3	cysteine desulfurase	iscS	404	45,235	5.8	106	40	cysteine metabolic process
117	*	549591826	U6W8M2	elongation factor Tu, partial	tuf	388	42,767	5.1	146	39	GTP catabolic process, translational elongation
118	*	549591826	U6W8M2	elongation factor Tu, partial	tuf	400	42,767	5.1	133	46	GTP catabolic process, translational elongation
118		525837571	S5HDU4	acetate kinase	ackA	388	43,572	5.9	121	49	acetyl-CoA biosynthetic process, phosphorylation, organic acid metabolic process
119	*	549591826	U6W8M2	elongation factor Tu, partial	tuf	388	42,767	5.1	72	27	GTP catabolic process, translational elongation

Table S1. *Cont.*

Spot		GI Number	UniProt AC	Protein Name	Gene Names	Protein Length	Protein M_W (Da)	pI Value	Mascot Score	MS Coverage	Biological Process
120	*	549591826	U6W8M2	elongation factor Tu, partial	tuf	388	42,767	5.1	140	39	GTP catabolic process, translational elongation
121	*	525840204	S5HJ10	2-amino-3-ketobutyrate CoA ligase		398	43,346	5.8	95	23	biosynthetic process
122	*	549591826	U6W8M2	elongation factor Tu, partial	tuf	388	42,767	5.1	128	45	GTP catabolic process, translational elongation
123	*	525838606	S5HGT3	glucose-1-phosphatase/inositol phosphatase		413	45,872	6.7	121	34	dephosphorylation
124	*	32699711	Q8ZQT5	Protein TolB Precursor	tolB	430	46,120	8.7	125	34	protein transport
125	*	32699711	Q8ZQT5	Protein TolB Precursor	tolB	430	46,120	8.7	100	29	protein transport
126	*	549591826	U6W8M2	elongation factor Tu, partial	tuf	388	42,767	5.1	188	48	GTP catabolic process, translational elongation
127	*	549591826	U6W8M2	elongation factor Tu, partial	tuf	388	42,767	5.1	164	43	GTP catabolic process, translational elongation
128	*	379657333	E1WCY8	glycerol-3-phosphate-binding periplasmic protein, partial	ugpB	430	47,567	6.6	149	35	transport
129		525837733	S5H3P6	fructose-bisphosphate aldolase		350	38,292	6.4	268	74	metabolic process
130		525841142	S5HEC3	isoaspartyl dipeptidase		390	40,584	5.6	196	55	proteolysis
131		525837618	S5H3B3	glycerophosphodiester phosphodiesterase	glpQ	356	40,456	5.6	123	36	glycerol metabolic process, lipid metabolic process
133	*	525838070	S5H221	ribose-phosphate pyrophosphokinase	prs	321	35,073	5	110	43	5-phosphoribose 1-diphosphate biosynthetic process, phosphorylation

Table S1. *Cont.*

Spot		GI Number	UniProt AC	Protein Name	Gene Names	Protein Length	Protein M_W (Da)	pI Value	Mascot Score	MS Coverage	Biological Process
134	*	549620658	U6YG44	ribose-phosphate pyrophosphokinase	prs	315	36,852	5.4	87	35	5-phosphoribose 1-diphosphate biosynthetic process, phosphorylation
134		525838167	S5H652	universal stress protein E		337	35,730	5	77	38	response to stress
135	*	525839322	S5H9F3	endo-1,4-D-glucanase	tsf	283	30,453	5	78	29	translational elongation
136	*	525839307	S5H9E2	acetyl-CoA carboxylase subunit alpha	accA	319	35,435	5.3	143	52	malonyl-CoA biosynthetic process, fatty acid biosynthetic process
137	*	525836836	S5HBS0	*N*-acetylneuraminate lyase	nanA	297	32,606	5.5	145	66	*N*-acetylneuraminate catabolic process, carbohydrate metabolic process
138		525837478	S5H2W5	deferrochelatase/ peroxidase YfeX		299	33,256	5.4	202	64	oxidation-reduction process
139	*	525837479	S5HB07	short-chain dehydrogenase		263	28,023	4.9	127	39	oxidation-reduction process
140		525838038	S5H4K8	cell division inhibitor MinD		270	29,596	5.2	138	39	barrier septum site selection, ATP catabolic process
141	*	545007466	Q8Z7S0	outer membrane protein A	ompA	350	37,606	5.5	137	40	conjugation, transmembrane ion transport
142		386730548	E8XLZ1	aminoglycoside resistance protein A	strA	272	30,474	4.7	77	36	response to antibiotic
143		386730548	E8XLZ1	aminoglycoside resistance protein A	strA	272	30,474	4.7	102	36	response to antibiotic
144	*	525839130	S5H505	ion channel protein Tsx		287	32,758	4.8	110	41	nucleoside transmembrane transport

Table S1. *Cont.*

Spot		GI Number	UniProt AC	Protein Name	Gene Names	Protein Length	Protein M_W (Da)	pI Value	Mascot Score	MS Coverage	Biological Process
148	*	558705045	P0A251	Chain E, wild type Salmonella alkyl hydroperoxide reductase C	ahpC	187	20,717	4.9	102	53	response to oxidative stress, oxidation-reduction process
149	*	525839663	S5H972	inorganic pyrophosphatase	ppa	176	19,778	4.9	112	43	phosphate-containing compound metabolic process
150	*	525840697	S5HD31	dTDP-4-dehydrorhamnose 3, 5-epimerase		182	20,577	4.9	106	42	lipopolysaccharide biosynthetic process
152	*	525838356	P0A2F5	superoxide dismutase [Fe]	sodB	193	21,352	5.5	121	55	oxidation-reduction process, superoxide metabolic process
153	*	525838604	S5HEA4	NAD(P)H:quinone oxidoreductase		198	20,854	5.8	141	53	negative regulation of transcription, DNA-templated, oxidation-reduction process
154		525832427	S5HED7	dihydropteroate synthase		271	28,452	5.9	223	78	folic acid-containing compound biosynthetic process, pteridine-containing compound metabolic process
155	*	158428843	P0A1F6	Chain B, unliganded crystal structure of uridine phosphorylase from *Salmonella* Typhimurium at 1.90 Å resolution	udp	253	27,162	6.1	166	62	UMP salvage, nucleotide catabolic process
156		525838717	S5H7R3	dimethyl sulfoxide reductase subunit B		205	23,677	6.8	141	40	

Table S1. *Cont.*

Spot		GI Number	UniProt AC	Protein Name	Gene Names	Protein Length	Protein M_W (Da)	pI Value	Mascot Score	MS Coverage	Biological Process
157	*	525838541	S5HGL3	3-ketoacyl-ACP reductase	fabG	244	25,586	7.5	134	54	fatty acid biosynthetic process, oxidation-reduction process
157	*	525838541	S5HGL3	3-ketoacyl-ACP reductase	fabG	244	25,586	7.5	134	54	fatty acid biosynthetic process, oxidation-reduction process
158		525838799	S5HEX7	amino acid ABC transporter substrate-binding protein	glnH	248	27,245	9	96	38	transport
159		525837935	S5H1P9	cystine transporter subunit		266	28,787	9.3	147	37	transport
160		257097748	Q7CQR3	Chain A, 1.2 Å crystal structure of glutaredoxin 2 (Grxb) from *Salmonella* Typhimurium in complex with glutathione	grxB	215	24,694	8.8	117	45	cell redox homeostasis
161	*	525839861	S5HKD9	50S ribosomal protein L1	rplA	234	24,713	10.1	113	50	translation
162	*	525840424	S5HJP0	30S ribosomal protein S3	rpsC	233	25,967	10.7	147	45	translation
163	*	383498061	H8M073	30S ribosomal protein S4	rpsD	202	23,024	10.4	179	53	translation
164	*	525840432	S5HCC1	50S ribosomal protein L6	rplF	177	18,905	10.2	97	37	translation
165	*	525840429	S5HJP4	50S ribosomal protein L5	rplE	179	20,362	9.9	189	86	translation
166	*	525837455	S5H0A2	transaldolase	tal	316	35,741	6	155	43	pentose-phosphate shunt
167		525836988	S5H1G3	oxidoreductase		294	31,590	6	170	49	oxidation-reduction process
169		525836802	S5H297	quinone oxidoreductase		324	34,537	6.5	122	36	oxidation-reduction process
170	*	383496004	H8M3B7	glyceraldehyde 3-phosphate dehydrogenase A	gapA	334	36,159	6.1	118	36	glucose metabolic process, oxidation-reduction process

Table S1. *Cont.*

Spot		GI Number	UniProt AC	Protein Name	Gene Names	Protein Length	Protein M_W (Da)	pI Value	Mascot Score	MS Coverage	Biological Process
171	*	525838077	S5H5U9	2-dehydro-3-deoxyphosphooctonate aldolase	kdsA	284	31,004	5.9	122	41	keto-3-deoxy-D-manno-octulosonic acid biosynthetic process
172	*	525837418	S5H2Q1	4-hydroxy-3-methylbut-2-en-1-yl diphosphate synthase	ispG	372	40,886	5.8	113	40	isopentenyl diphosphate biosynthetic process, methylerythritol 4-phosphate pathway, oxidation-reduction process
173	*	525838428	S5H5P5	phenylalanyl-tRNA synthetase	PheS	327	36,789	5.8	226	53	phenylalanyl-tRNA aminoacylation
176	*	525839611	S5HJM0	multifunctional aminopeptidase A	pepA	503	55,368	7	161	29	proteolysis, protein metabolic process
177		525840290	S5H897	glutathione reductase		450	49,004	5.7	75	24	glutathione metabolic process, cell redox homeostasis
178	*	525836874	S5H9A9	phosphoglucosamine mutase	glmM	445	47,697	5.7	99	26	carbohydrate metabolic process
179	*	525837501	S5HDL8	glutaminyl-tRNA synthetase	gltX	471	53,999	5.5	170	35	glutamyl-tRNA aminoacylation
180	*	525840371	S5HLS6	phosphoenolpyruvate carboxykinase	pckA	539	59,895	5.6	133	33	gluconeogenesis, phosphorylation
181		525839536	S5HJD9	heme ABC transporter ATP-binding protein		555	62,385	5.4	167	33	ATP catabolic process
182	*	525839059	S5HFM4	5'-nucleotidase	ushA	550	60,557	5.8	106	16	dephosphorylation, nucleotide catabolic process
183	*	525838245	S5H2K8	malate dehydrogenase	maeA	565	63,255	5.1	170	36	oxidation-reduction process, malate metabolic process

Table S1. *Cont.*

Spot	GI Number	UniProt AC	Protein Name	Gene Names	Protein Length	Protein M_W (Da)	pI Value	Mascot Score	MS Coverage	Biological Process
184	525838711	S5HH39	keto-acid formate acetyltransferase		760	85,293	5.7	279	55	carbohydrate metabolic process
185	549725641	U4MED2	transketolase	tktA	648	70,745	5.2	96	25	metabolic process
186	525838711	S5HH39	keto-acid formate acetyltransferase		760	85,293	5.7	158	25	carbohydrate metabolic process

* an orthologous protein was also identified using the *Salmonella* spp Swiss-Prot database.

Table S2. *Salmonella* Typhimurium SL1344 proteins identified by MALDI-TOF MS.

Spot		GI Number	UniProt AC	Protein Name	Gene Names	Protein Length	Protein M_W (Da)	pI Value	Mascot Score	MS Coverage	Biological Process
200	*	525838467	S5H702	glyceraldehyde-3-phosphate dehydrogenase		331	35,735	6.4	304	79	glucose metabolic process, oxidation-reduction process
201	*	525839731	S5HK16	molecular chaperone GroEL	groEL	548	57,421	4.7	332	61	protein folding, protein refolding
202	*	549591826	U6W8M2	elongation factor Tu, partial	tuf	388	42,767	5.1	315	73	GTP catabolic process, translational elongation
203	*	545007466	Q8Z7S0	outer membrane protein A	ompA	350	37,606	5.5	253	58	conjugation, transmembrane ion transport
204	*	116489820	A0FLL2	phase I flagellin middle domain variant C12	fliC	386	40,713	4.6	282	67	ciliary or bacterial-type flagellar motility
205	*	525839621	S5HJN2	arginine deiminase	arcA	406	45,875	5.4	308	69	protein citrullination
206	*	383495039	H8M4U4	30S ribosomal protein S2	rpsB	236	26,310	6.7	178	60	translation
207	*	525839509	S5HGY3	molecular chaperone DnaK	dnaK	638	69,273	4.7	338	57	response to stress, protein folding
208		525838703	S5H6I4	30S ribosomal protein S1	rpsA	557	61,250	4.7	337	57	translation
209	*	525841024	S5HLB3	heat shock protein 90	htpG	624	71,442	5	509	70	protein folding, response to stress
210		525839734	S5HHN2	aspartate ammonia-lyase	aspA	478	52,880	5	230	41	tricarboxylic acid cycle, aspartate metabolic process
211	*	525840415	S5H8L9	elongation factor G	fusA	704	77,722	5	515	69	GTP catabolic process, translational elongation
212		525837616	S5HDZ5	*sn*-glycerol-3-phosphate dehydrogenase subunit A	glpA	542	59,819	6.2	524	69	glycerol-3-phosphate metabolic process, polyol catabolic process, oxidation-reduction process
213	*	525837154	S5HA44	enolase	Eno	432	45,627	5.1	301	68	glycolysis
214	*	383497733	H8M1H4	phosphoglycerate kinase	pgk	382	40,719	4.9	316	72	glycolysis, phosphorylation

Table S2. *Cont.*

Spot		GI Number	UniProt AC	Protein Name	Gene Names	Protein Length	Protein M_W (Da)	pI Value	Mascot Score	MS Coverage	Biological Process
215	*	525839623	S5H935	ornithine carbamoyltransferase		334	37,021	5.2	190	50	ornithine metabolic process
216		525837618	S5H3B3	glycerophosphodiester phosphodiesterase	glpQ	356	40,456	5.6	223	68	glycerol metabolic process, lipid metabolic process
217	*	525837344	S5HAQ1	protein disaggregation chaperone		857	95,549	5.2	539	62	response to heat
218		525839720	S5H6L3	fumarate reductase flavoprotein subunit		596	66,021	5.9	330	51	electron transport chain, oxidation-reduction process
219		525839720	S5H6L3	fumarate reductase flavoprotein subunit		596	66,021	5.9	396	54	electron transport chain, oxidation-reduction process
220	*	525840371	S5HLS6	phosphoenolpyruvate carboxykinase	pckA	539	59,895	5.6	290	56	gluconeogenesis, phosphorylation
221	*	525839909	S5HI67	glycerol kinase	glpK	502	56,301	5.3	358	62	glycerol-3-phosphate metabolic process, phosphorylation, carbohydrate metabolic process
222	*	525837615	S5H0R2	glycerol-3-phosphate dehydrogenase subunit B	glpB	419	46,213	5.5	188	49	glycerol catabolic process
223	*	549591826	U6W8M2	elongation factor Tu, partial	tuf	388	42,767	5.1	320	67	GTP catabolic process, translational elongation
224	*	532641272	T5JVY4	phosphoglyceromutase, partial	gpmA	243	27,736	5.4	203	67	glycolysis
225		525839721	S5HK04	fumarate reductase iron-sulfur subunit		244	27,784	6.1	121	43	tricarboxylic acid cycle
226	*	158428843	P0A1F6	Chain B, unliganded crystal structure of uridine phosphorylase from *Salmonella* Typhimurium at 1.90 Å resolution	Udp	253	27,162	6.1	220	74	UMP salvage, nucleotide catabolic process

Table S2. *Cont.*

Spot	GI Number	UniProt AC	Protein Name	Gene Names	Protein Length	Protein M_W (Da)	pI Value	Mascot Score	MS Coverage	Biological Process
227	525839622	S5HA86	carbamate kinase		310	33,558	5.4	214	45	arginine metabolic process
228	525838718	S5H6K1	dimethyl sulfoxide reductase subunit A		814	91,201	6.4	365	42	oxidation-reduction process
229	525839098	S5H7L3	peptidase	lon	784	87,698	6	197	31	response to stress, proteolysis, misfolded or incompletely synthesized protein catabolic process
230	525838094	S5HCW3	acetaldehyde dehydrogenase		892	96,668	6.2	247	49	alcohol metabolic process, carbon utilization, oxidation-reduction process
231	525838094	S5HCW3	acetaldehyde dehydrogenase		892	96,668	6.2	93	17	alcohol metabolic process, carbon utilization, oxidation-reduction process
232 *	525839101	S5HI58	trigger factor	tig	432	48,037	4.7	393	64	protein peptidyl-prolyl isomerization, cell cycle, protein transport
233 *	525837491	S5HDK7	phosphoenolpyruvate-protein phosphotransferase		575	63,557	4.6	140	40	phosphoenolpyruvate-dependent sugar phosphotransferase system, phosphorylation
234 *	525840077	S5HBD9	F_0F_1 ATP synthase subunit beta	AtpD	460	50,309	4.8	166	52	plasma membrane ATP synthesis coupled proton transport, ATP hydrolysis coupled proton transport
235 *	525840042	S5HBA0	transcription termination factor Rho	rho	419	47,021	6.9	125	41	ATP catabolic process, DNA-templated transcription, termination

Table S2. *Cont.*

Spot		GI Number	UniProt AC	Protein Name	Gene Names	Protein Length	Protein M_W (Da)	pI Value	Mascot Score	MS Coverage	Biological Process
236	*	525838467	S5H702	glyceraldehyde-3-phosphate dehydrogenase		331	35,735	6.4	212	54	glucose metabolic process, oxidation-reduction process
237		525840348	S5HBC4	glycerol-3-phosphate dehydrogenase	glpD	502	57,117	6.5	189	34	glycerol-3-phosphate metabolic process
238		525840348	S5HBC4	glycerol-3-phosphate dehydrogenase	glpD	502	57,117	6.5	328	49	glycerol-3-phosphate metabolic process
239	*	525838327	S5H6N7	fumarate hydratase		548	60,734	6.3	354	62	generation of precursor metabolites and energy
240		525837426	S5HDD9	inosine 5'-monophosphate dehydrogenase	guaB	488	52,201	6.2	220	49	purine nucleotide biosynthetic process
241		525839380	S5H5Q6	dihydrolipoamide dehydrogenase		474	50,893	5.8	234	52	cell redox homeostasis
242	*	525840075	S5H7K0	F_0F_1 ATP synthase subunit alpha	atpA	513	55,307	5.6	357	57	ATP hydrolysis coupled proton transport, plasma membrane ATP synthesis coupled proton transport
243	*	549591826	U6W8M2	elongation factor Tu, partial	tuf	388	42,767	5.1	209	51	GTP catabolic process, translational elongation
244	*	383497733	H8M1H4	phosphoglycerate kinase	pgk	382	40,719	4.9	287	61	glycolysis, phosphorylation
245		525837064	S5H9U4	fructose-bisphosphate aldolase		359	39,360	5.6	75	26	glycolysis
246	*	525839393	S5H8G1	guanosine 5'-monophosphate oxidoreductase	guaC	347	37,514	6.1	173	41	oxidation-reduction process
247		525837733	S5H3P6	fructose-bisphosphate aldolase		350	38,292	6.4	292	73	metabolic process
248	*	525838611	S5HGT7	DNA-binding protein	cbpA	306	34,672	6.6	175	48	protein folding

Table S2. *Cont.*

Spot		GI Number	UniProt AC	Protein Name	Gene Names	Protein Length	Protein M_W (Da)	pI Value	Mascot Score	MS Coverage	Biological Process
249		532641030	T5K5Z8	peptidyl-prolyl *cis-trans* isomerase, partial		263	28,015	8.7	193	49	protein peptidyl-prolyl isomerization
250	*	525838914	S5HF78	glucosamine-6-phosphate deaminase	nagB	266	29,784	6.4	139	58	*N*-acetylglucosamine metabolic process
251		525838341	S5HG46	glutathionine *S*-transferase		201	22,543	6.1	169	76	metabolic process
252	*	525839546	S5HJF3	purine nucleoside phosphorylase	deoD	239	26,189	5.5	247	84	purine nucleoside metabolic process
253	*	414021944	P0A2F5	superoxide dismutase [Fe]	sodB	193	21,380	5.5	129	55	oxidation-reduction process, superoxide metabolic process
254	*	525839322	S5H9F3	endo-1,4-D-glucanase	tsf	283	30,453	5	259	56	translational elongation
255	*	525839322	S5H9F3	endo-1,4-D-glucanase	tsf	283	30453	5	188	49	translational elongation
256	*	525838240	S5H2K1	outer membrane porin protein C		362	39,655	4.5	105	42	transmembrane ion transport
257	*	529293148	not mapped	DNA-directed RNA polymerase subunit beta, partial			141,946	5.1	203	17	
258	*	525836878	S5H169	translation initiation factor IF-2	infB	892	97,514	5.8	103	17	GTP catabolic process, translational initiation
259	*	525837582	S5H4B4	NADH dehydrogenase subunit G		908	100,877	5.7	77	9	ATP synthesis coupled electron transport
260		525838094	S5HCW3	acetaldehyde dehydrogenase		892	96,668	6.2	245	40	alcohol metabolic process, carbon utilization, oxidation-reduction process
261		525838094	S5HCW3	acetaldehyde dehydrogenase		892	96,668	6.2	204	39	alcohol metabolic process, carbon utilization, oxidation-reduction process

Table S2. *Cont.*

Spot		GI Number	UniProt AC	Protein Name	Gene Names	Protein Length	Protein M_W (Da)	pI Value	Mascot Score	MS Coverage	Biological Process
262		525838094	S5HCW3	acetaldehyde dehydrogenase		892	96,668	6.2	255	47	alcohol metabolic process, carbon utilization, oxidation-reduction process
263		525838094	S5HCW3	acetaldehyde dehydrogenase		892	96,668	6.2	296	48	alcohol metabolic process, carbon utilization, oxidation-reduction process
264	*	525840102	S5HBG1	DNA gyrase subunit B	gyrB	804	90,125	5.7	165	25	DNA topological change, ATP catabolic process
265		525838441	S5HGB6	hydroperoxidase II	katE	750	83,802	5.7	329	35	hydrogen peroxide catabolic process, oxidation-reduction process
266	*	525840415	S5H8L9	elongation factor G	fusA	704	77,722	5	248	40	GTP catabolic process, translational elongation
267	*	525840415	S5H8L9	elongation factor G	fusA	704	77,722	5	352	60	GTP catabolic process, translational elongation
268		525839377	S5H9J9	bifunctional aconitate hydratase 2/2-methylisocitrate dehydratase		865	94,040	5.1	208	31	tricarboxylic acid cycle
269	*	81521548	Q8ZLT3	polyribonucleotide nucleotidyltransferase	Pnp	711	77,048	4.9	139	16	RNA phosphodiester bond hydrolysis, exonucleolytic, mRNA catabolic process
270	*	525839315	S5H5L1	outer membrane protein assembly factor YaeT	bamA	804	89,585	4.8	328	43	Gram-negative-bacterium-type cell outer membrane assembly, protein insertion into membrane
271		525838417	S5H6V2	phosphoenolpyruvate synthase		792	87,610	4.8	143	21	pyruvate metabolic process
272		525839382	S5H9K2	pyruvate dehydrogenase	aceE	887	99,802	5.4	273	35	oxidation-reduction process
273	*	525837456	S5HDG7	malic enzyme		759	82,726	5.4	271	41	malate metabolic process

Table S2. *Cont.*

Spot		GI Number	UniProt AC	Protein Name	Gene Names	Protein Length	Protein M_W (Da)	pI Value	Mascot Score	MS Coverage	Biological Process
274	*	525837570	S5H0M0	phosphate acetyltransferase		714	77,572	5.2	302	51	acetyl-CoA biosynthetic process
275		525838898	S5H714	ornithine decarboxylase		732	83,253	5.6	311	57	cellular amino acid metabolic process
276		525838733	S5H6L9	ATP-dependent Clp protease ATP-binding protein	clpA	758	84,156	5.8	331	50	ATP catabolic process, proteolysis
277		525839720	S5H6L3	fumarate reductase flavoprotein subunit		596	66,021	5.9	199	33	electron transport chain, oxidation-reduction process
278		525839720	S5H6L3	fumarate reductase flavoprotein subunit		596	66,021	5.9	265	42	electron transport chain, oxidation-reduction process
279	*	525838912	S5H878	glutaminyl-tRNA synthetase	glnS	555	64,068	5.6	173	29	glutamyl-tRNA aminoacylation, glutaminyl-tRNA aminoacylation
280	*	525838719	S5HEM6	seryl-tRNA synthetase	serS	430	48,835	5.3	149	41	seryl-tRNA aminoacylation, selenocysteinyl-tRNA(Sec) biosynthetic process
282		525836996	S5HC51	hydrogenase 2 large subunit		567	62,912	5.8	180	36	oxidation-reduction process
283	*	525839990	S5H7C8	proline dipeptidase	pepQ	443	50,309	5.8	215	42	proteolysis
284		525838742	S5H7T6	pyruvate dehydrogenase		572	62,214	6.3	108	15	metabolic process
285		525838959	S5HFC4	citrate lyase subunit alpha		509	55,103	6.3	138	24	acetyl-CoA metabolic process
286	*	54041937	P67557	glucans biosynthesis protein G, precursor	mdoG	511	57,808	8.8	181	40	glucan biosynthetic process
287		525840348	S5HBC4	glycerol-3-phosphate dehydrogenase	glpD	502	57,117	6.5	372	53	glycerol-3-phosphate metabolic process
288	*	383496004	H8M3B7	glyceraldehyde 3-phosphate dehydrogenase A	gapA	334	36,159	6.1	115	31	glucose metabolic process, oxidation-reduction process

Table S2. *Cont.*

Spot		GI Number	UniProt AC	Protein Name	Gene Names	Protein Length	Protein M_W (Da)	pI Value	Mascot Score	MS Coverage	Biological Process
289	*	525839438	S5H8K5	peptidyl-prolyl cis-trans isomerase	surA	428	47,221	6.9	205	44	protein transport
290		525836915	S5GYP6	L-serine dehydratase		454	48,957	6	195	45	gluconeogenesis
291	*	525837388	S5H2M8	serine hydroxymethyltransferase	glyA	417	45,597	6.0	155	42	cellular amino acid biosynthetic process, methylation, one-carbon metabolic process
292		525837137	S5H337	L-serine dehydratase		455	49,449	5.8	124	21	gluconeogenesis
293	*	549591826	U6W8M2	elongation factor Tu, partial	tuf	388	42,767	5.1	226	53	GTP catabolic process, translational elongation
294	*	549591826	U6W8M2	elongation factor Tu, partial	tuf	388	42,767	5.1	134	36	GTP catabolic process, translational elongation
295	*	549591826	U6W8M2	elongation factor Tu, partial	tuf	388	42,767	5.1	241	53	GTP catabolic process, translational elongation
296	*	525839508	S5H8T7	molecular chaperone DnaJ	dnaJ	379	41,857	9.3	202	49	response to stress
297	*	549591826	U6W8M2	elongation factor Tu, partial	tuf	388	42,767	5.1	180	51	GTP catabolic process, translational elongation
298	*	379657333	E1WCY8	glycerol-3-phosphate-binding periplasmic protein, partial	ugpB	430	47,567	6.6	211	52	transport
299	*	525838606	S5HGT3	glucose-1-phosphatase/inositol phosphatase		413	45,872	6.7	178	44	dephosphorylation
300		525838351	S5HG53	*N*-ethylmaleimide reductase		365	39,455	6.2	274	82	oxidation-reduction process
301		525841142	S5HEC3	isoaspartyl dipeptidase		390	40,584	5.6	206	63	proteolysis
302	*	525839927	S5HAZ7	6-phosphofructokinase	pfkA	320	35,235	5.5	135	51	fructose 6-phosphate metabolic process
303		525840287	S5HC17	phosphosugar isomerase		324	36,406	5.9	87	22	carbohydrate metabolic process

Table S2. *Cont.*

Spot		GI Number	UniProt AC	Protein Name	Gene Names	Protein Length	Protein M_W (Da)	pI Value	Mascot Score	MS Coverage	Biological Process
304		525840371	S5HLS6	phosphoenolpyruvate carboxykinase	pckA	539	59,895	5.6	228	49	gluconeogenesis, phosphorylation
305	*	525837455	S5H0A2	transaldolase	tal	316	35,741	6	100	29	pentose-phosphate shunt
305		525840389	S5HJK6	tryptophanyl-tRNA synthetase	trpS	334	37,605	6	93	35	tryptophanyl-tRNA aminoacylation
306	*	525836819	S5H969	malate dehydrogenase	mdh	312	32,626	6	244	76	malate metabolic process, carbohydrate metabolic process, tricarboxylic acid cycle, oxidation-reduction process
307		383496518	H8M6D2	PTS system mannose-specific transporter subunit IIAB	gptB	300	32,631	5.8	241	63	phosphoenolpyruvate-dependent sugar phosphotransferase system
308	*	525836836	S5HBS0	*N*-acetylneuraminate lyase	nanA	297	32,606	5.5	145	62	*N*-acetylneuraminate catabolic process, carbohydrate metabolic process
309	*	525838135	S5H284	enoyl-ACP reductase		262	27,971	5.5	110	44	fatty acid biosynthetic process, oxidation-reduction process
310		525837478	S5H2W5	deferrochelatase/ peroxidase YfeX		299	33,256	5.4	103	41	oxidation-reduction process
311	*	414022212	K8SG73	DNA-binding transcriptional regulator PhoP		223	25,486	5.2	268	80	phosphorelay signal transduction system, transcription, DNA-templated
312	*	525839914	S5HI71	triosephosphate isomerase	tpiA	255	27,071	5.6	150	47	gluconeogenesis, glycolysis, pentose-phosphate shunt
313	*	525838604	S5HEA4	NAD(P)H:quinone oxidoreductase		198	20,854	5.8	128	53	negative regulation of transcription, DNA-templated, oxidation-reduction process

Table S2. *Cont.*

Spot		GI Number	UniProt AC	Protein Name	Gene Names	Protein Length	Protein M_W (Da)	pI Value	Mascot Score	MS Coverage	Biological Process
314		525839140	S5H512	peroxiredoxin		200	22,417	5.1	148	68	oxidation-reduction process
315	*	525841256	P0A2C6	D-ribose-binding periplasmic protein	rbsB	296	30,943	9.2	184	61	chemotaxis, carbohydrate transport
316	*	383495039	H8M4U4	30S ribosomal protein S2	rpsB	236	26,310	6.7	112	46	translation
317		525838717	S5H7R3	dimethyl sulfoxide reductase subunit B		205	23,677	6.8	81	30	
318		525838566	S5HGP3	glutaredoxin		215	24,562	8.9	148	68	cell redox homeostasis
319		525837935	S5H1P9	cystine transporter subunit		266	28,787	9.3	182	41	transport
320		525838799	S5HEX7	amino acid ABC transporter substrate-binding protein	glnH	248	27,245	9	107	43	transport
321	*	525838541	S5HGL3	3-ketoacyl-ACP reductase	fabG	244	25,586	7.5	148	54	fatty acid biosynthetic process
322	*	525839320	S5H5L3	ribosome recycling factor	frr	185	20,600	9	116	51	translational termination
323	*	808038	P43019	Mn-superoxide dismutase	sodA	206	22,952	6.1	94	50	superoxide metabolic process
324	*	525840887	B5F8E4	UPF0227 protein YcfP	ycfP	180	21,292	6.3	134	52	
325	*	525839863	S5H9T4	transcription antitermination protein NusG	nusG	181	20,532	6.4	148	61	transcription antitermination
326	*	525839861	S5HKD9	50S ribosomal protein L1	rplA	234	24,713	10.1	119	50	translation
327	*	525840420	S5H8M3	50S ribosomal protein L4	rplD	201	22,073	10.2	110	51	translation
328	*	525840429	S5HJP4	50S ribosomal protein L5	RplE	179	20,362	9.9	192	72	translation
329		525836802	S5H297	quinone oxidoreductase		324	34,537	6.5	159	50	oxidation-reduction process
330	*	378447564	C9XCD0	quinone oxidoreductase		323	34,816	6.8	187	52	oxidation-reduction process
331	*	525839917	S5HAZ0	aldolase		291	32,007	6	220	75	metabolic process
332		525836994	S5H9L4	hydrogenase		328	36,837	6.7	153	36	
333	*	525837545	S5H0J2	acetyl-CoA carboxylase subunit beta	accD	304	33,536	8.9	121	32	malonyl-CoA biosynthetic process
334	*	383498081	H8M093	50S ribosomal protein L2	rplB	255	27,917	11.4	103	33	translation

Table S2. *Cont.*

Spot		GI Number	UniProt AC	Protein Name	Gene Names	Protein Length	Protein M_W (Da)	pI Value	Mascot Score	MS Coverage	Biological Process
335	*	525839514	S5HGY9	transaldolase	tal	317	35,320	5	304	77	carbohydrate metabolic process, pentose-phosphate shunt
336	*	549620658	U6YG44	ribose-phosphate pyrophosphokinase	prs	337	36,852	5.4	100	43	5-phosphoribose 1-diphosphate biosynthetic process, phosphorylation
337	*	525840441	S5HLY6	DNA-directed RNA polymerase subunit alpha	rpoA	329	36,717	4.8	288	56	transcription, DNA-templated
338		525838502	S5H738	isocitrate dehydrogenase		416	46,101	5	161	42	glyoxylate cycle, tricarboxylic acid cycle, oxidation-reduction process
339	*	525839547	S5HA11	phosphopentomutase	deoB	407	44,558	5	195	44	cellular metabolic compound salvage, 5-phosphoribose 1-diphosphate biosynthetic process, deoxyribonucleotide catabolic process
340	*	525837775	S5H1A5	lipopolysaccharide biosynthesis protein		437	48,530	5.1	223	45	metabolic process
341	*	525839621	S5HJN2	arginine deiminase	arcA	406	45,875	5.4	159	57	protein citrullination
342	*	383498339	H8LYI4	2,3-bisphosphoglycerate-independent phosphoglycerate mutase	GpmI	523	57,355	4.9	170	31	glycolysis
343		378443762	C9X823	aminoacyl-histidine dipeptidase precursor		485	52,693	5	193	52	proteolysis
344		545007139	T2K3A1	phosphoglucomutase	pgm	531	56,752	5.5	129	27	carbohydrate metabolic process
345	*	525837089	S5H9X0	lysyl-tRNA synthetase	lysS	505	57,596	4.9	273	52	lysyl-tRNA aminoacylation
346	*	525840293	S5HB85	oligopeptidase A		680	77,237	5	154	32	proteolysis
347	*	545007761	T2K502	pyruvate kinase	pykF	452	48,907	5.4	162	44	glycolysis, phosphorylation

Table S2. *Cont.*

Spot		GI Number	UniProt AC	Protein Name	Gene Names	Protein Length	Protein M_W (Da)	pI Value	Mascot Score	MS Coverage	Biological Process
348	*	525837153	S5H1Y1	CTP synthetase	pyrG	545	60,540	5.6	248	35	*de novo* CTP biosynthetic process, glutamine metabolic process
349	*	525839673	S5H982	3'-nucleotidase	cpdB	647	70,587	5.6	191	41	dephosphorylation
350	*	312913650	U3SMX6	protein disaggregation chaperone		857	95,531	5.2	128	23	response to heat
351	*	525837976	S5HEZ9	aspartyl-tRNA synthetase	aspS	590	65,910	5.3	185	39	aspartyl-tRNA aminoacylation
352	*	525838245	S5H2K8	malate dehydrogenase	maeA	565	63,255	5.1	241	51	oxidation-reduction process, malate metabolic process
353		525836996	S5HC51	hydrogenase 2 large subunit		567	62,912	5.8	180	36	oxidation-reduction process
354		525839748	S5H9G5	fumarate hydratase		548	60,523	5.9	141	35	generation of precursor metabolites and energy
355	*	383498724	H8M0S2	pyridine nucleotide-disulfide oxidoreductase	sthA	444	49,878	6.2	160	40	NADP metabolic process, cell redox homeostasis, hydrogen ion transmembrane transport
357	*	525837953	S5H4C9	trehalose-6-phosphate synthase		473	53,662	6.2	109	28	trehalose biosynthetic process
358	*	525837464	S5HAZ4	aldehyde dehydrogenase		467	49,428	6.2	102	23	metabolic process
360		525840290	S5H897	glutathione reductase		450	49,004	5.7	72	19	glutathione metabolic process, cell redox homeostasis
361	*	525837399	S5HAU3	cysteine desulfurase	iscS	404	45,235	5.8	177	39	cysteine metabolic process
362	*	549591826	U6W8M2	elongation factor Tu, partial	tuf	388	42,767	5.1	165	48	GTP catabolic process, translational elongation
363	*	525837571	S5HDU4	acetate kinase	ackA	400	43,572	5.9	178	58	acetyl-CoA biosynthetic process, phosphorylation, organic acid metabolic process
364	*	549591826	U6W8M2	elongation factor Tu, partial	tuf	388	42,767	5.1	180	49	GTP catabolic process, translational elongation

Table S2. *Cont.*

Spot		GI Number	UniProt AC	Protein Name	Gene Names	Protein Length	Protein M_W (Da)	pI Value	Mascot Score	MS Coverage	Biological Process
365	*	549591826	U6W8M2	elongation factor Tu, partial	tuf	388	42,767	5.1	179	44	GTP catabolic process, translational elongation
366	*	549591826	U6W8M2	elongation factor Tu, partial	tuf	388	42,767	5.1	137	39	GTP catabolic process, translational elongation
367	*	549591826	U6W8M2	elongation factor Tu, partial	tuf	388	42,767	5.1	212	48	GTP catabolic process, translational elongation
368	*	525838428	S5H5P5	phenylalanyl-tRNA synthetase	pheS	327	36,789	5.8	220	51	phenylalanyl-tRNA aminoacylation
369	*	525837493	S5H2X9	cysteine synthase A		323	34,571	5.8	156	55	cysteine biosynthetic process from serine
370	*	525839354	S5HGI9	pantoate-beta-alanine ligase	panC	284	31,952	5.8	148	40	pantothenate biosynthetic process
371		525836988	S5H1G3	oxidoreductase		294	31,590	6	149	51	oxidation-reduction process
373	*	525839412	S5H9N6	16S rRNA methyltransferase	rsmH	313	34,754	6.7	90	28	rRNA base methylation
377	*	525839019	S5HFH9	methenyltetrahydrofolate cyclohydrolase	folD	288	31,109	6.1	128	37	one-carbon metabolic process
378	*	525838456	S5HGD0	exonuclease III		268	30,937	6.2	151	41	DNA catabolic process, exonucleolytic
379	*	525838077	S5H5U9	2-dehydro-3-deoxyphosphooctonate aldolase	kdsA	284	31,004	5.9	197	53	keto-3-deoxy-D-manno-octulosonic acid biosynthetic process
381		525840658	S5HC50	carbohydrate degradation protein		276	30,549	5.7	106	48	
382		525832427	S5HED7	dihydropteroate synthase		271	28,452	5.9	199	74	folic acid-containing compound biosynthetic process, pteridine-containing compound metabolic process

Table S2. *Cont.*

Spot		GI Number	UniProt AC	Protein Name	Gene Names	Protein Length	Protein M_W (Da)	pI Value	Mascot Score	MS Coverage	Biological Process
384		525839520	S5H629	transcriptional regulator		238	27,388	5.2	125	45	phosphorelay signal transduction system, transcription, DNA-templated
385		525838038	S5H4K8	cell division inhibitor MinD		270	29,596	5.2	169	53	barrier septum site selection, ATP catabolic process
386	*	545007466	Q8Z7S0	outer membrane protein A	ompA	350	37,606	5.5	194	51	conjugation, transmembrane ion transport
387	*	525837479	S5HB07	short-chain dehydrogenase		263	28,023	4.9	102	26	oxidation-reduction process
388	*	554075122	V1JW55	trehalose-6-phosphate synthase		437	49,334	6.1	73	13	trehalose biosynthetic process
389		414021469	K8SL93	GTP-binding protein YchF	ychF	363	39,947	4.7	166	52	ATP catabolic process
390	*	428698020	P0A264	Chain A, loop deletion mutant of *Salmonella* Typhi osmoporin (Ompc), an outer membrane protein	ompC	378	37,246	4.3	96	38	ion transmembrane transport
391	*	549591826	U6W8M2	elongation factor Tu, partial	tuf	388	42,767	5.1	215	48	GTP catabolic process, translational elongation
392	*	525837076	S5HCC5	glycine cleavage system aminomethyltransferase T	gcvT	364	40,305	5.2	131	32	methylation
393	*	525837769	S5HBY3	dTDP-glucose 4,6-dehydratase		361	40,865	5.5	146	42	nucleotide-sugar metabolic process
394		525838194	S5HD60	hypothetical protein CFSAN001921_08980		447	51,275	5.5	149	37	
395		383496375	H8M5H8	periplasmic murein peptide-binding protein MppA	mppA	525	58,570	7.7	164	35	transport
396	*	532641168	K8VWI5	serine endoprotease		442	46,224	9.3	108	29	proteolysis
398	*	32699711	Q8ZQT5	protein TolB	tolB	430	46,120	8.7	181	43	protein transport
399		414011741	K8RUH9	plasmid partition protein B		324	36,755	8.8	73	28	

Table S2. *Cont.*

Spot		GI Number	UniProt AC	Protein Name	Gene Names	Protein Length	Protein M_W (Da)	pI Value	Mascot Score	MS Coverage	Biological Process
400	*	525839832	S5HAS7	B12-dependent methionine synthase	metH	1227	136,660	4.8	75	14	methionine biosynthetic process
401	*	525838427	S5H6W4	phenylalanyl-tRNA synthetase	pheT	795	87,940	5	120	15	phenylalanyl-tRNA aminoacylation
402		525839382	S5H9K2	pyruvate dehydrogenase	aceE	887	99,802	5.4	232	31	oxidation-reduction process
405		525838094	S5HCW3	acetaldehyde dehydrogenase		892	96,668	6.2	146	30	alcohol metabolic process, carbon utilization, oxidation-reduction process
406	*	383497556	Q8VQB5	Cell invasion protein SipA	sipA	670	72,588	6.1	77	16	pathogenesis
407	*	525837794	S5HC05	thiosulfate reductase		758	83,488	7.9	114	20	oxidation-reduction process
408	*	525837794	S5HC05	thiosulfate reductase		758	83,488	7.9	110	20	oxidation-reduction process
409	*	554021358	V1FLU5	carboxy-terminal protease		660	74,470	6.2	86	21	proteolysis
412		525839720	S5H6L3	fumarate reductase flavoprotein subunit		596	66,021	5.9	239	41	electron transport chain, oxidation-reduction process
414		525838470	S5H396	MltA-interacting protein MipA		248	27,975	5.4	79	43	
416	*	525839130	S5H505	ion channel protein Tsx		287	32,758	4.8	108	41	nucleoside transmembrane transport

* an orthologous protein was also identified using the *Salmonella* spp Swiss-Prot database.
